# Insulin-like growth factor receptor signaling in breast tumor epithelium protects cells from endoplasmic reticulum stress and regulates the tumor microenvironment

**DOI:** 10.1186/s13058-018-1063-2

**Published:** 2018-11-20

**Authors:** Alison E. Obr, Sushil Kumar, Yun-Juan Chang, Joseph J. Bulatowicz, Betsy J. Barnes, Raymond B. Birge, Deborah A. Lazzarino, Emily Gallagher, Derek LeRoith, Teresa L. Wood

**Affiliations:** 1 Department of Pharmacology, Physiology & Neuroscience, Rutgers-New Jersey Medical School, Cancer Institute of New Jersey, Newark, NJ 07101 USA; 2 Department of Microbiology, Biochemistry & Molecular Genetics, Rutgers-New Jersey Medical School, Cancer Institute of New Jersey, Newark, NJ 07101 USA; 3 Office of Advance Research Computing, Rutgers-New Jersey Medical School, Newark, NJ 07102 USA; 4 Feinstein Institute for Medical Research, Northwell Health, Manhasset, NY 11030 USA; 5 Division of Endocrinology, Diabetes and Bone Diseases, The Samuel Bronfman Department of Medicine, Icahn Sinai School of Medicine at Mt. Sinai, New York, NY 10029 USA

**Keywords:** IGF-1R, IL-6, CCL2, Breast cancer, Wnt1, Cellular stress, MMP

## Abstract

**Background:**

Early analyses of human breast cancer identified high expression of the insulin-like growth factor type 1 receptor (IGF-1R) correlated with hormone receptor positive breast cancer and associated with a favorable prognosis, whereas low expression of IGF-1R correlated with triple negative breast cancer (TNBC). We previously demonstrated that the IGF-1R acts as a tumor and metastasis suppressor in the Wnt1 mouse model of TNBC. The mechanisms for how reduced IGF-1R contributes to TNBC phenotypes is unknown.

**Methods:**

We analyzed the METABRIC dataset to further stratify IGF-1R expression with patient survival and specific parameters of TNBC. To investigate molecular events associated with the loss of IGF-1R function in breast tumor cells, we inhibited IGF-1R in human cell lines using an IGF-1R blocking antibody and analyzed MMTV-Wnt1-mediated mouse tumors with reduced IGF-1R function through expression of a dominant-negative transgene.

**Results:**

Our analysis of the Molecular Taxonomy of Breast Cancer International Consortium (METABRIC) dataset revealed association between low IGF-1R and reduced overall patient survival. IGF-1R expression was inversely correlated with patient survival even within hormone receptor-positive breast cancers, indicating reduced overall patient survival with low IGF-1R was not due simply to low IGF-1R expression within TNBCs. Inhibiting IGF-1R in either mouse or human tumor epithelial cells increased reactive oxygen species (ROS) production and activation of the endoplasmic reticulum stress response. IGF-1R inhibition in tumor epithelial cells elevated interleukin (IL)-6 and C-C motif chemokine ligand 2 (CCL2) expression, which was reversed by ROS scavenging. Moreover, the *Wnt1/dnIGF-1R* primary tumors displayed a tumor-promoting immune phenotype. The increased CCL2 promoted an influx of CD11b^+^ monocytes into the primary tumor that also had increased matrix metalloproteinase (MMP)-2, MMP-3, and MMP-9 expression. Increased MMP activity in the tumor stroma was associated with enhanced matrix remodeling and collagen deposition. Further analysis of the METABRIC dataset revealed an increase in IL-6, CCL2, and MMP-9 expression in patients with low IGF-1R, consistent with our mouse tumor model and data in human breast cancer cell lines.

**Conclusions:**

Our data support the hypothesis that reduction of IGF-1R function increases cellular stress and cytokine production to promote an aggressive tumor microenvironment through infiltration of immune cells and matrix remodeling.

**Electronic supplementary material:**

The online version of this article (10.1186/s13058-018-1063-2) contains supplementary material, which is available to authorized users.

## Background

Triple-negative breast cancers (TNBCs), classified as estrogen receptor (ER)-negative, progesterone receptor (PR)-negative and lacking human epidermal growth factor receptor 2 (HER2) amplification remain the most aggressive breast tumor subtype, and approximately 50% of TNBCs classify as basal-like [1]. While chemotherapy treatment for TNBC primary tumors is effective short-term, tumors ultimately recur and frequently metastasize [2, 3]. Hyperactivation of the Wnt pathway is common in breast carcinomas where it is often activated in the absence of downstream mutations [4], and Wnt1 overexpression in mammary epithelium is sufficient to form basal-like tumors in mice with low metastatic potential [5].

Signaling through the insulin-like growth factor type 1 receptor (IGF-1R) is complex, and defining its role in breast tumorigenesis has been controversial. Early studies reported that expression of the IGF-1R correlated with ER expression and predicted a favorable phenotype [6]. Numerous studies have further confirmed cross-talk between the ER and IGF-1R in breast cancer (for review, see [7]). Consistent with these data, loss of IGF-1R has been associated with breast tumor progression into a more undifferentiated phenotype [8], suggesting that IGF-1R is involved in tumor suppression. However, other findings have shown that IGF signaling is a positive mediator of breast cancer growth and survival (for reviews, see [9, 10]). Since IGF signaling promotes tumor cell proliferation and survival, various inhibitors have been developed to attenuate IGF signaling (for reviews, see [11, 12]). Despite early preclinical findings, the usefulness of disrupting IGF-1R signaling in clinical trials has been less than promising and, in some cases, inhibiting the pathway has led to worse outcomes [11, 12]. Collectively, these diverse results support the possibility that the IGF-1R has a dual function as both a tumor suppressor and an oncogene.

In our recent studies, we established a mouse line with transgenic expression of a kinase-dead, dominant-negative IGF-1R (*MMTV-dnIGF-1R*) in combination with *MMTV-Wnt1* expression to test how decreased IGF-1R signaling in the mammary epithelium impacts a well-established mouse model of basal-like breast cancer [5]. Attenuation of IGF-1R in this model resulted in decreased tumor latency, an enhanced basal phenotype, and potentiation of lung metastases (Additional file 1: Table S1, see also [1]). These results were surprising given that the *MMTV-Wnt1* tumors have low metastatic potential [5]. However, similar findings were reported from conditionally deleting IGF-1R in a prostate cancer mouse model [13]. These data are also consistent with new reports that have correlated high IGF-1R and ERα expression in luminal B breast tumors with a better prognosis [14]. Recent queries of the Cancer Genome Atlas (TCGA) database for IGF-1R expression identified higher IGF-1R expression in luminal A and luminal B breast tumors and lower expression in HER2-like and triple-negative tumors [15]. Taken together, these data suggest the function of IGF-1R is dependent on the tumor type and signaling context.

Several studies have established that IGF signaling is important for maintaining cellular stress homeostasis such that modifications in IGF signaling result in alterations in stress signaling. Endoplasmic reticulum (EnR) stress is a consequence of increased misfolded proteins and results in the production of reactive oxygen species (ROS) and ultimately cell death (for reviews, see [16, 17]. Reduction-of-function mutations in the IGF signaling pathway in *Caenorhabditis elegans* result in activation of the unfolded protein response (UPR) leading to an enhanced EnR stress response [18]. Furthermore, activation of IGF-1 signaling in breast cancer and neuronal cells protects from EnR-stress-induced apoptosis by enhancing EnR stress responses to promote cellular adaptability for cell survival maintenance [19, 20]. Moreover, the inhibition of IGF signaling in breast cancer cells results in activation of EnR stress to induce autophagy and protect from apoptosis [21]. These results suggest the IGF pathway protects cells from EnR stress, and that perturbation of the IGF pathway leads to enhanced overall EnR stress.

In the present study, we tested the hypothesis that attenuated IGF-1R function promotes tumor epithelial cell stress resulting in tumor stromal environment alterations to establish an aggressive phenotype in breast tumors. We determined that IGF-1R is essential in tumor suppression in breast tumorigenesis. We demonstrate that attenuated IGF-1R signaling in the *MMTV-Wnt1* mouse mammary tumor model and in human breast cancer cell lines increases tumor epithelial cellular stress, resulting in upregulation of cytokine production. These changes result in altered migration and infiltration of tumor immune cells and dramatic alterations in the tumor microenvironment associated with promoting primary tumor epithelial cell extravasation.

## Methods

### Antibodies and reagents

Rabbit monoclonal anti-phospho-eIF2a (D9G8), rabbit monoclonal anti-eukaryotic initiation factor 2-alpha (eIF2a) (D7D3), rabbit monoclonal anti-protein disulfide isomerase (PDI) (C81H6), mouse monoclonal anti-C/EBP homologous protein (CHOP) (L63F7), rabbit monoclonal anti-phospho-Akt (Ser473) (D9E), rabbit monoclonal anti-Akt (11E7), rabbit monoclonal anti-phospho-IGF-1R/IR (D6D5L), and rabbit monoclonal anti-IGF-1R (D23H3) antibodies were purchased from Cell Signaling. Rabbit polyclonal anti-matrix metalloproteinase (MMP)-2 (ab37150) and anti-MMP-9 (ab38898) antibodies were purchased from Abcam. Mouse monoclonal anti-β-actin (A5441) was purchased from Sigma Aldrich. IMC-A12 (10 mg/ml), a monoclonal antibody against IGF-1R, was provided by ImClone Systems, a wholly owned subsidiary of Eli Lilly and Co. Human IgG antibody (31154; 11.3 mg/ml), a monoclonal antibody used as a control, was purchased from Invitrogen. N-acetyl-L-cysteine (A9165) was purchased from Sigma Aldrich.

### Animal models

All animal protocols were approved by the Rutgers University Institutional Animal Care and Use Committee (Newark, NJ, USA). All experiments were managed in accord with the National Institutes of Health (NIH) guidelines for the care and use of laboratory animals. The *MMTV-Wnt1* line on an FVB background (FVB.Cg-Tg(Wnt1)1Hev/J) was obtained as a gift from Dr Yi Li. The *MMTV-Wnt1//MMTV-dnIgf1r* line was described previously [1]. Female littermates (*MMTV-Wnt1/dnIGF-1R*; homozygous for *dnIGF-1R* versus *MMTV-Wnt1*) were used for experiments. Tumors were harvested when they reached 1.5 cm^3^.

### Cell lines

The human MCF7 breast cancer cell line was provided by Dr Robert Wieder (Rutgers University-NJMS) and validated with the human short tandem repeat (STR) profiling cell authentication service (American Type Culture Collection (ATCC)). The RAW264.7 mouse monocyte cell line was purchased from ATCC. All cells were maintained at 37 °C and 5% CO_2_ and in either MEM (MCF7) or DMEM (RAW264.7) medium supplemented with 10% FBS (Sigma Aldrich) and 100 U/ml of penicillin and streptomycin.

### Detection of reactive oxygen species and cellular stress

Fresh tumors were drop-fixed in methacarn (60% methanol, 30% chloroform, 10% glacial ascetic acid) overnight at 4 °C, embedded in paraffin, and sectioned at 7 μm. Tumor sections were processed for OxyIHC™ with antigen retrieval, chemical derivatization of protein carbonyl groups with 2,4-dinitrophenylhydrazine (DNPH), and incubation with dinitrophenyl (DNP) moiety-specific primary antibody (1:100) overnight at 4 °C according to the manufacturer’s protocol (Millipore, S7450). Negative controls for each tumor section were processed without primary antibody and with derivatization control solution. Positive detection of protein oxidation was obtained using 3,3′ diaminobenzidine (DAB) and counterstained with hematoxylin.

MCF7 cells were stained with 2′,7′-dichlorofluorescin diacetate (DCFDA) reagent (20 μM, Abcam) according to the manufacturer’s protocol, washed, and treated with human IgG or IMC-A12 (100 nM) at varying time points in 1X Supplemental Buffer. MCF7 cells were treated with Tert-Butyl Hydrogen Peroxide (TBHP) (100 μM) for 4 h as a positive control. DCFDA levels were measured with the Perkin Elmer Victory3V plate reader using the Excitation 485/Emission 535 filter.

To inhibit ROS production, MCF7 cells were treated with N-acetyl cysteine (NAC, 5 mM) for 1 h before treatment with either human IgG or A12 (100 nM). Cells were harvested after 24 h in TRI Reagent (Sigma Aldrich) for RNA isolation and qRT-PCR analysis.

### Protein isolation, immunoblotting, and ELISA detection

Snap-frozen tumor pieces were pulverized using liquid nitrogen. Pulverized tumor pieces and cells from cell lines were lysed with radioimmunoprecipitation assay (RIPA) buffer. Lysates containing equal amounts of total protein were boiled in 2X Laemmli buffer (BioRad) and electrophoresed on 4–12% polyacrylamide sodium dodecyl sulfate (SDS) gels (Invitrogen). Separated proteins were transferred to nitrocellulose membranes, incubated with anti-phospho-eIF2a (1:1000), anti-eIF2a (1:1000), and anti-ß-actin (1:5000) for cell-line lysates or anti-CHOP (1:1000), anti-PDI (1:1000), and anti-ß-actin (1:5000) for whole-tumor lysates, and antibody binding to protein bands was detected by enhanced chemiluminescence (Amersham).

Whole tumor lysates in RIPA buffer were incubated with the human IL-6 Quantikine ELISA kit (R&D Systems) according to the manufacturer’s protocol. Levels of IL-6 were detected using the Perkin Elmer Victory3V plate reader at an absorbance of 450 nm.

### RNA isolation and quantitative real-time PCR by targeted pathway arrays

Whole tumor RNA was extracted using TRI Reagent (Sigma Aldrich) according to manufacturer’s protocol. For the cytokine/chemokine targeted array provided by Dr Sophia Ran, RNA from five tumors was reverse transcribed separately and the cytokine/chemokine array was performed and analyzed using the ∆∆ cycle threshold (Ct) method as previously described [22] to determine expression of *MMTV-Wnt1/dnIGF-1R* compared to *MMTV-Wnt1* tumors by fold change. For the Wnt signaling pathway targeted array (Qiagen RT^2^ Profiler Array, PAMM-043Z), 1 μg of RNA from five tumors was pooled and reverse transcribed using the RT2 easy first strand synthesis kit (Qiagen) per manufacturer’s protocol. The array was performed using the BioRad CFX96 real-time PCR machine and the RT2 SYBR Green qPCR mastermix (Qiagen) per manufacturer’s protocol. Analysis was performed using the ∆∆Ct method with the RT^2^ Profiler PCR Array Data Analysis Suite (Qiagen).

### Analysis of tumor immune cells by flow cytometry

Tumor immune cells isolated as described above were resuspended at 10^6^ cells/100 μl in FACS buffer (2% BSA, 2% goat serum in PBS). Cells were immunolabeled with the following fluorochrome-conjugated cell surface antibodies: anti-CD45 PE/Cy5 (0.25 μg/100 μl), anti-CD11b PerCP (0.25 μg/100 μl), anti-CD4 FITC (0.25 μg/100 μl), anti-CD8 PE (0.25 μg/100 μl), anti-CD25 PE/Cy7 (0.5 μg/100 μl), and anti-FOXP3 Alexa Fluor 647 (5 μl/100 μl) all purchased from BioLegend. Single cells were prepared for flow cytometry as previously described [23] or according to the manufacturer’s protocol (BioLegend, for FOXP3). Cell-associated fluorescence was acquired and analyzed using the BD LSR II cytometer and TreeStar Inc. FlowJo software, respectively.

### Mammary epithelial cell dissociation for flow sorting and flow cytometry

Mammary tumor epithelial cells (MECs) were isolated from *MMTV-Wnt1* or *MMTV-Wnt1/dnIGF-1R* mice similarly to our prior study [1]. Whole tumors were excised and dissociated with the gentleMACs tissue dissociator (130–093-235, protocol m_TDK2) and mouse specific tumor dissociation kit (Miltenyi, 130–096-730). Organoids that retain basement membrane attachments were trypsinized (0.05% Trypsin-EDTA, Gibco) and filtered with a 40-μm cell strainer (BD Biosciences) to isolate a single cell suspension of dissociated tumor MECs. Isolated tumor MECs were counted using a hemocytometer prior to flow cytometry or sorting.

Mammary tumor immune cells were isolated from tumors as described previously [23]. Whole tumors were excised, minced, and digested with Collagenase-I (10 U/ml), Collagenase-IV (400 U/ml; Worthington), and DNase-1 (30 mg/ml; Sigma Aldrich) for 25 min at 37 °C. Cells from digested tumors were filtered with a 70-μm cell strainer (BD Biosciences) and pelleted. Red blood cells were lysed with an erythrocyte lysis buffer (150 mM Ammonium chloride, 1 mM Potassium bicarbonate, 130 μM EDTA, pH 7.2) for 2 min, filtered with a 70-μm cell strainer (BD Biosciences) and pelleted. Isolated immune cells were counted using a hemocytometer prior to flow cytometry or magnetic bead sorting.

### Sorting of mammary tumor epithelial and immune cells

Tumor MECs from either *MMTV-Wnt1* or *MMTV-Wnt1/dnIGF-1R* mice (*n* = 5) were isolated for single cells as described above and resuspended at 10^6^ cells/ml in FACS buffer (2% BSA, 2% goat serum in PBS). Cells were immunolabeled with fluorochrome-conjugated cell surface antibodies as described in our previous studies [1]. Single cells were prepared for FACS as previously described [24] and sorted at 70 psi using a 70-um nozzle on the Beckton Dickenson FACS Vantage directly into RLT Buffer (Qiagen, 79216) for RNA isolation and qRT-PCR analysis.

Tumor immune cells from individual tumors (*n* = 4 per genotype) were isolated for single cells as described above and resuspended at 10^7^ cells in MACS BSA Stock Solution according to the manufacturer’s protocol (Miltenyi, 130–091-376). Cells were immunolabeled with rat monoclonal anti-CD11b antibody conjugated to magnetic microbeads (Miltenyi, 130–049-601). Immuno-labeled cells were run through a magnetic column according to the manufacturer’s protocol (Miltenyi); both CD11b negative and positive flow-through were collected. Cells were resuspended in RLT Buffer (Qiagen) for RNA isolation and qRT-PCR analysis.

### RNA isolation and real-time quantitative PCR

For sorted tumor epithelial and immune cells, RNA was extracted and purified according to the manufacturer’s protocol (Qiagen). Whole tumor and human cell line RNA was extracted using TRI Reagent (Sigma Aldrich) according to the manufacturer’s protocol. RNA concentration and quality were assayed by absorbance (A_230_, A_260_, A_280_) with the NanoDrop ND-1000 (Thermo Scientific). Complementary DNA (cDNA) was transcribed according to the manufacturer’s protocol using SuperScript II (Invitrogen) from total RNA (200 ng sorted cells, 1000 ng whole tumor and cell lines). Samples were run in technical triplicate to determine relative gene expression by real-time quantitative PCR (qRT-PCR) detected with SsoAdvanced Universal SYBR Green Supermix (BioRad) using the BioRad CFX96 real-time PCR machine according to the manufacturer’s instructions. Transcript levels were normalized to glyceraldehyde-3-phosphate dehydrogenase (GAPDH, for mouse) or ß-actin (for human), and data were analyzed using the Q-Gene software (BioTechniques Software Library) [25]. Primer oligonucleotide pairs for qRT-PCR are provided in Additional file 2: Table S2.

### Knockdown of IL-6 and CCL2 expression by small interfering RNA (siRNA)

SMARTpool ON-TARGET plus siRNA for IL-6 (L-043739-00-005) and CCL2 (L-042243-00-0005) and for scramble siGENOME Control Pool Non-Targeting #2 (Scr, D-001206-14-05) were resuspended (5 μM) using 1X siRNA buffer as described by the manufacturer’s protocol (Dharmacon). MCF7 cells treated with human IgG or IMC-A12 (100 nM) for 24 h were transfected with Scr, IL-6, or CCL2 siRNA (25 ng) using transfection reagent DharmaFECT 1 per manufacturer’s protocol (Dharmacon). Cells were harvested after 24 h with Accutase (Sigma Aldrich) for monocyte migration assays or in TRI Reagent (Sigma Aldrich) for RNA isolation and qRT-PCR analysis.

### Measurement of monocyte migration in real time

Cell migration of a monocyte cell line (RAW264.7) was performed using a real-time, label-free monitoring system (xCELLigence RTCA DP, Acea Biologicals) that measures micro-impedance of electrical current. MCF7 cells were seeded in the lower chamber of a CIM-16 plate (Acea Biologicals) at 20,000 cells/180 μl in MEM supplemented with 10% FBS and treated with human IgG or IMC-A12 (100 nM) for 24 h. RAW264.7 monocytes were serum-starved (DMEM supplemented with 0.1% FBS) overnight and seeded in the CIM-16 plate upper chamber at 40,000 cells/100 μl after a medium-only baseline reading was taken. The migration rate is plotted as delta cell index (electrical impendence change) over 48 h (data point every 10 min).

MCF7 cells treated with human IgG or IMC-A12 (100 nM) for 24 h and transfected with siScr control, siIL-6, or siCCL2 and seeded in the lower chamber of a CIM-16 plate (Acea Biosciences) as described above. Serum-starved (DMEM supplemented with 0.1% FBS overnight) RAW264.7 monocytes were seeded in the CIM-16 plate upper chamber and a baseline reading was taken as described above. The impedance data were acquired and plotted as described above.

### Histology and immunofluorescence

Tumor tissues (*n* = 4 per genotype) were drop-fixed in 4% paraformaldehyde (PFA), embedded in paraffin, and sectioned at 7 μm. Tumor sections were used for hematoxylin and eosin or Masson’s Trichrome staining, or further processed for antigen retrieval for immunofluorescence (IF) as described previously [26]. Masson’s Trichrome staining was performed according to the manufacturer’s protocol (Abcam). Tissue sections were immunostained with primary antibodies - MMP-2 (1:50) or MMP-9 (1:50) - and with species-specific fluorochrome-conjugated secondary antibodies (1:500, Invitrogen). All IF sections were stained with 4',6-diamidino-2-phenylindole (DAPI) (1:10,000) to visualize cell nuclei.

### Tissue *in situ* zymography

*In situ* zymography was performed as described previously [27]. Tumor tissues (*n* = 4 per genotype) were drop-fixed in IHC Zinc Fixative (BD Pharmingen) for 24 h at 4 °C, embedded in paraffin, and sectioned at 8 μm. The DQ gelatin substrate (Invitrogen) was incubated with tissue sections as previously described [27].

### Image capture

An Olympus Provis AX70 brightfield/fluorescent microscope attached to the QIClick QImaging camera with iVision Mac scientific imaging processing software (4.0.16, BioVision Technologies) was used to capture images from histological and immunofluorescence staining. At least five individual fields were captured from tumor sections at × 10 or × 20 magnification (*n* = 4 per genotype).

### Molecular Taxonomy of Breast Cancer International Consortium (METABRIC) dataset analysis

METABRIC data [28] downloaded from cBioPortal [29, 30] were used for the analysis, with expression values and associated clinical data generated from 1982 patients. The *z* scores comprised 299 patients with TNBC and 990 patients with ER+/PR+ breast cancer. Gene expression levels in individual patients were identified from the METABRIC dataset using the *z* scores identified from cBioPortal. Selected gene expression values of patients with TNBC or ER+/PR+ cancer, and different prediction analysis of microarray 50 (PAM50) identified subtypes were analyzed using the independent two-sample *t* test. Logistic regression was used to examine the distribution of expression values between each cancer type group and to estimate the probability of one subtype versus another subtype based on observed expression values. The Kaplan–Meier survival curve generated from the METABRIC dataset was plotted using the Package “survival” within the R environment. The log-rank test was performed to test the statistical difference between survival functions of the sub-grouped patients. All these calculations were conducted within the R environment, R version 3.3.2.

### Statistics

All graphical data were expressed as the mean ± SEM. Statistical comparisons were carried out using GraphPad Prism6 software. Student’s *t* test was used for two-group comparisons. One-way analysis of variance (ANOVA) with Bonferroni’s post-hoc test was used for multiple treatment comparisons. Specific comparisons are described in the figure legends when necessary. Power calculations were performed based on pilot data to determine the number of tumor samples necessary. For immune flow cytometry, a standard deviation of 27% and an approximate normal distribution of the data were assumed. Using the two-sample, two-sided *t* test to detect a significant difference with α = 0.025 and 80% power, suggested nine animals per group were needed. For qRT-PCR, a two-sided hypothesis test and α = 0.0025 and 80% power, indicated four animals per group for detectable differences.

## Results

### Low IGF-1R expression is associated with triple-negative, basal-like breast cancer and reduced overall survival

**Fig. 1:**
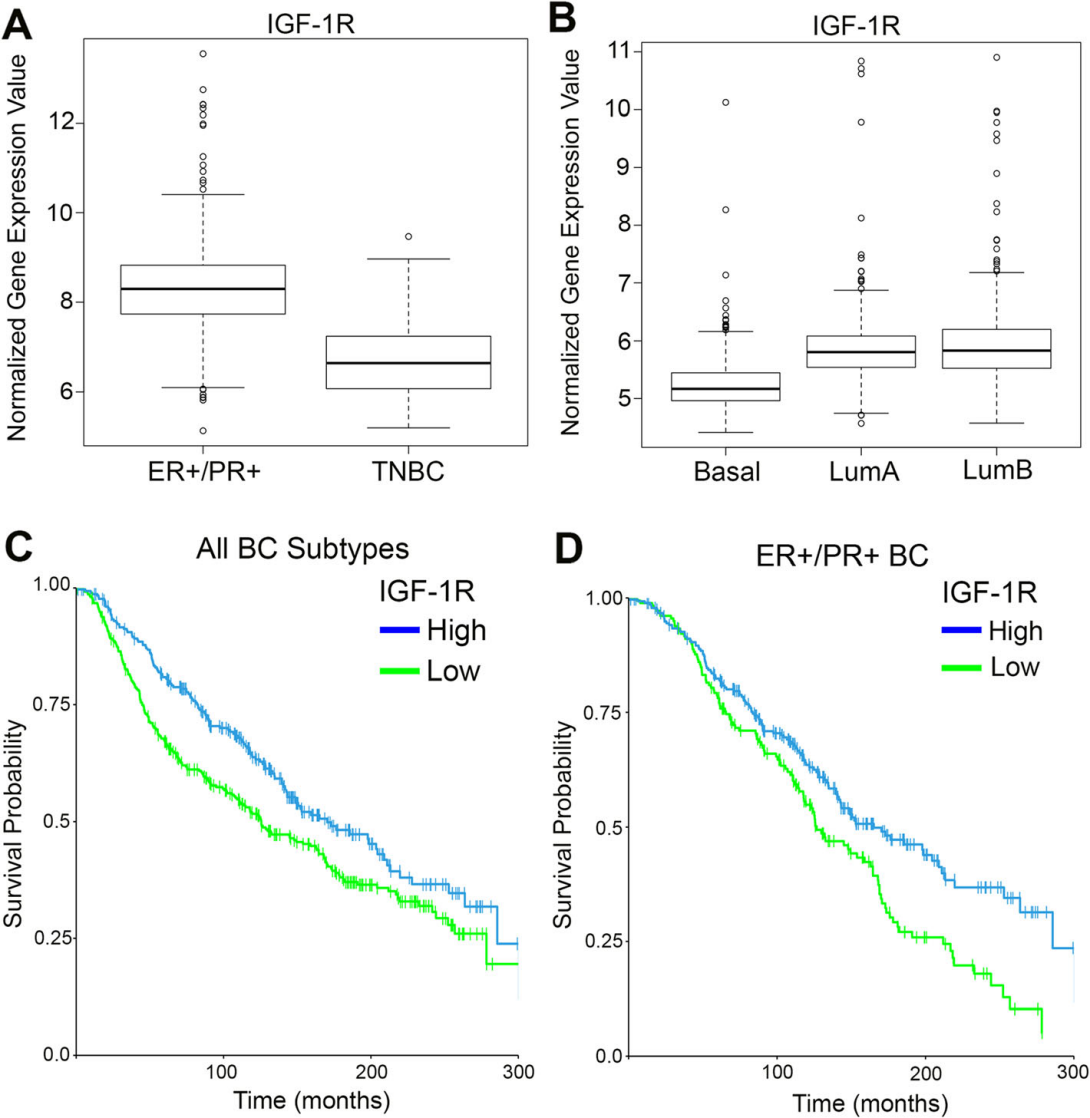
Decreased insulin-like growth factor type1 receptor (IGF-1R) expression correlates with basal-like, triple-negative breast cancer (TNBC). **a**, **b** Analysis of IGF-1R expression from the Molecular Taxonomy of Breast Cancer International Consortium (METABRIC) dataset. (**a**) Boxplot representation of IGF-1R expression levels in estrogen receptor (ER+)/progesterone receptor (PR+) breast cancer compared to TNBC (Student’s *t* test, *P* < 2.2 × 10^− 16^). (**b**) Boxplot representation of IGF-1R expression levels in the prediction analysis of microarray 50 (PAM50) subtypes: basal-like, luminal A (LumA), or luminal B (LumB) (Student’s *t* test, basal-like versus LumA *P* < 2.2 × 10^− 16^; basal-like versus LumB *P* < 2.2 × 10^− 16^). **c**, **d** Kaplan–Meier survival plot from analysis of the METABRIC dataset of overall survival in patients with low IGF-1R compared to high IGF-1R expression in all breast cancers (**c**) (log-rank test, *P* = 0.0021) and only ER+/PR+ breast cancer (**d**) (log-rank test, *P* = 0.0018). BC, breast cancer

Recent analysis of TCGA database for IGF-1R expression demonstrated IGF-1R expression levels are reduced in TNBC [15]. To further stratify the expression level of IGF-1R in breast cancers, we queried the METABRIC dataset that includes a larger patient sample size. Similar to Farabaugh et al. [15], IGF-1R expression was significantly lower in TNBC compared to ER+/PR+ breast cancer (Fig. 1a). Moreover, IGF-1R expression was lower in poorly differentiated, more aggressive basal-like breast cancers compared to the differentiated luminal A and luminal B subtypes as defined by PAM50 analysis (Fig. 1b). Importantly, analysis from the METABRIC dataset of patient overall survival in both ER+/PR+ breast cancer and TNBC revealed low expression of IGF-1R significantly correlates with worse overall survival compared to high expression of IGF-1R (Fig. 1c). Further stratification of ER+/PR+ patients confirmed worse overall survival correlates with low IGF-1R compared to high IGF-1R expression, even within this breast cancer subtype (Fig. 1d). Taken together, these data support the hypothesis that IGF-1R functions as a tumor suppressor in human breast cancer, and that reduced IGF-1R expression is detrimental to patient outcome.

### Tumor epithelial cell stress is activated by loss of IGF signaling

**Fig. 2:**
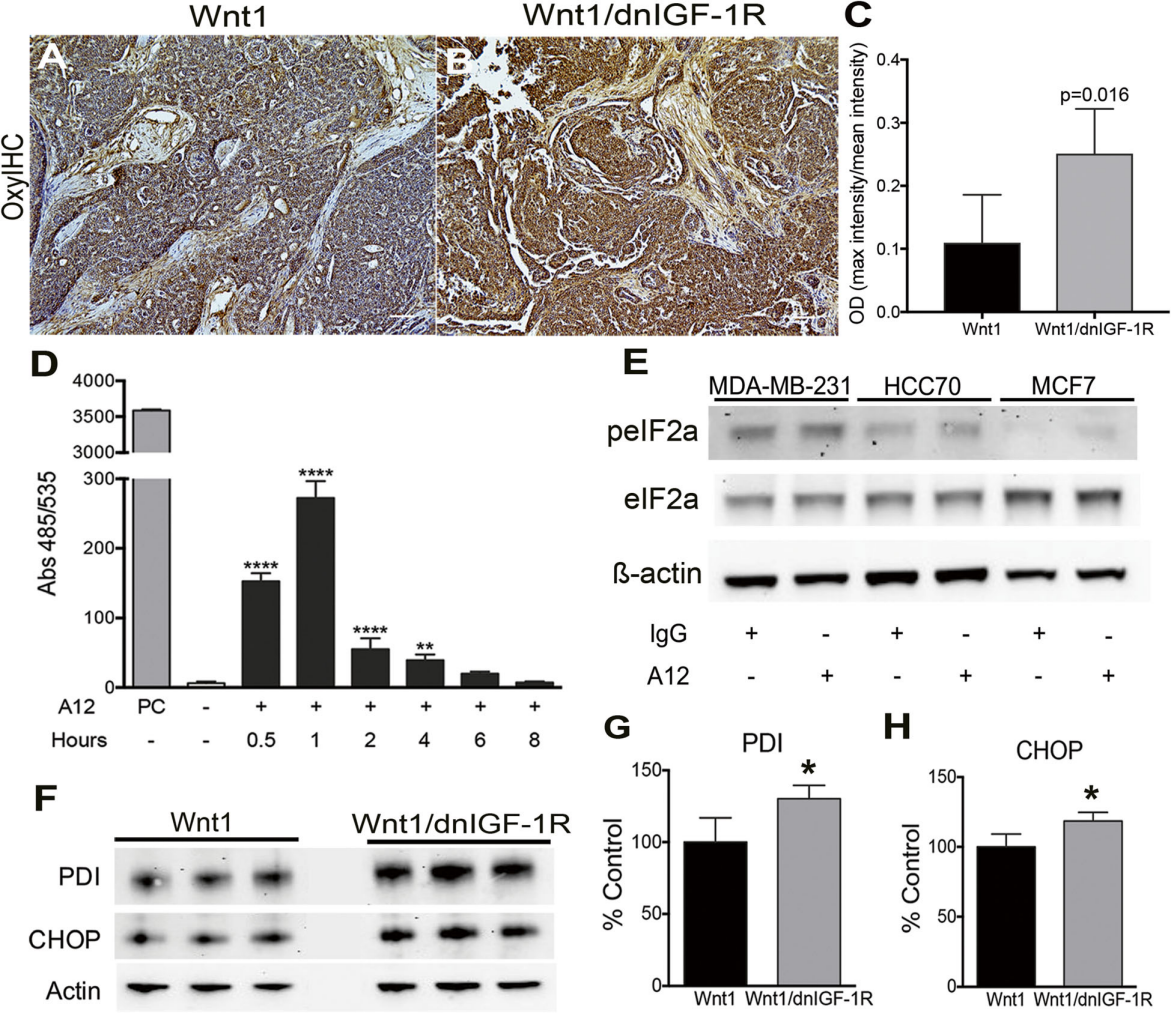
Cellular stress is increased in response to reduced insulin-like growth factor type1 receptor (IGF-1R) function. **a**, **b** Representative images showing OxyIHC-stained sections from *Wnt1* (**a**) or *Wnt1/dnIGF-1R* (**b**) tumors. **c** Quantification of OxyIHC-stained sections by 3,3′-diaminobenzidine intensity measurement (*n* = 5 tumor sections per genotype) (Student’s *t* test, *P* = 0.016). **d** The 2′,7′-dichlorofluorescin diacetate assay analysis of untreated or A12 treated MCF7 cells at subsequent time points: 0.5, 1, 2, 4, 6, and 8 h. PC (tert-butyl hydrogen peroxide positive control; 100 μM) (one-way analysis of variance, untreated versus A12: **P < 0.01, **** *P* < 0.0001; *n* = 3, with three technical replicates per experiment). **e** Representative western immunoblot showing levels of phospho-eukaryotic initiation factor 2-alpha (eIF2α) and total eIF2α protein compared to loading control (ß-actin) in untreated (IgG) and human breast cancer cell lines (MDA-MB-231, HCC70, MCF7) treated with A12 for 24 h. **f**, **h** Western immunoblotting showing levels of protein disulfide isomerase (PDI) and C/EBP homologous protein (CHOP) in *Wnt1* and *Wnt1/dnIGF-1R* tumors (**f**). Densitometry analysis of PDI (**g**) and CHOP (**h**) protein expression normalized to ß-actin in *Wnt1* and *Wnt1/dnIGF-1R* tumors. (Student’s *t* test*,* *P < 0.05; *n* = 3)

The IGF signaling pathway plays an important role in maintaining cellular stress homeostasis for cell proliferation and survival [19, 31, 32]. Previous studies from Novosyadlyy, et al. [19] established that activation of the IGF signaling pathway maintains EnR stress homeostasis by augmenting the adaptive capacity of the EnR and protecting against EnR-stress-induced apoptosis in MCF7 cells. Previous studies demonstrated activation of EnR stress signaling by increased generation of reactive oxygen species (ROS), which can be produced directly from the EnR through protein disulfide isomerase (PDI) [17]. Based on these and other studies linking EnR stress with tumorigenesis [18, 21, 33], we tested whether loss of IGF signaling in tumor epithelium increases EnR stress in breast tumor epithelial cells. Accumulation of ROS results in the addition of carboxyl groups to newly translated proteins [34]. We detected increased immunostaining for carboxyl groups in *MMTV-Wnt1/dnIGF-1R* compared to *MMTV-Wnt1* tumors (Fig. 2a-c). We further measured changes in ROS production with the DCFDA assay in MCF7 cells and observed increased fluorescence from 30 min to 4 h after IGF-1R inhibition with an IGF-1R blocking antibody, IMC-A12 (A12) (Fig. 2d). MCF7, a luminal human breast cancer cell line, has high levels of IGF-1R whereas HCC70, a basal-like breast cancer cell line, has intermediate levels of IGF-1R, and MDA-MB-231, a mesenchymal-like TNBC line, has low levels of IGF-1R relative to each other [35, 36]. Blocking IGF-1R in MCF7 cells with a monoclonal inhibiting antibody (A12) results in reduced Akt and IGF-1R phosphorylation after IGF-1 treatment as well as reduced total IGF-1R as expected due to receptor internalization and degradation (Additional file 3: Figure S1) [37]. Phosphorylation of eIF2α, a downstream effector of the EnR stress response, was increased in human breast cancer cell lines, HCC70 and MCF7, but not MDA-MB-231 after IGF-1R inhibition (Fig. 2e), while total eIF2α was unchanged, suggesting inhibition of IGF signaling activates EnR stress signaling in breast cell lines with low basal EnR stress. Moreover, PDI and CHOP, downstream targets of eIF2α activation, were increased in *MMTV-Wnt1/dnIGF-1R* tumors (Fig. 2f-h). Taken together, these data demonstrate that reduction of IGF signaling in tumor epithelial cells both in vivo and in vitro results in enhanced cellular stress through production of ROS and activation of the EnR stress pathway.

### Cellular stress with reduced IGF-1R signaling induces cytokine and chemokine expression

**Fig. 3:**
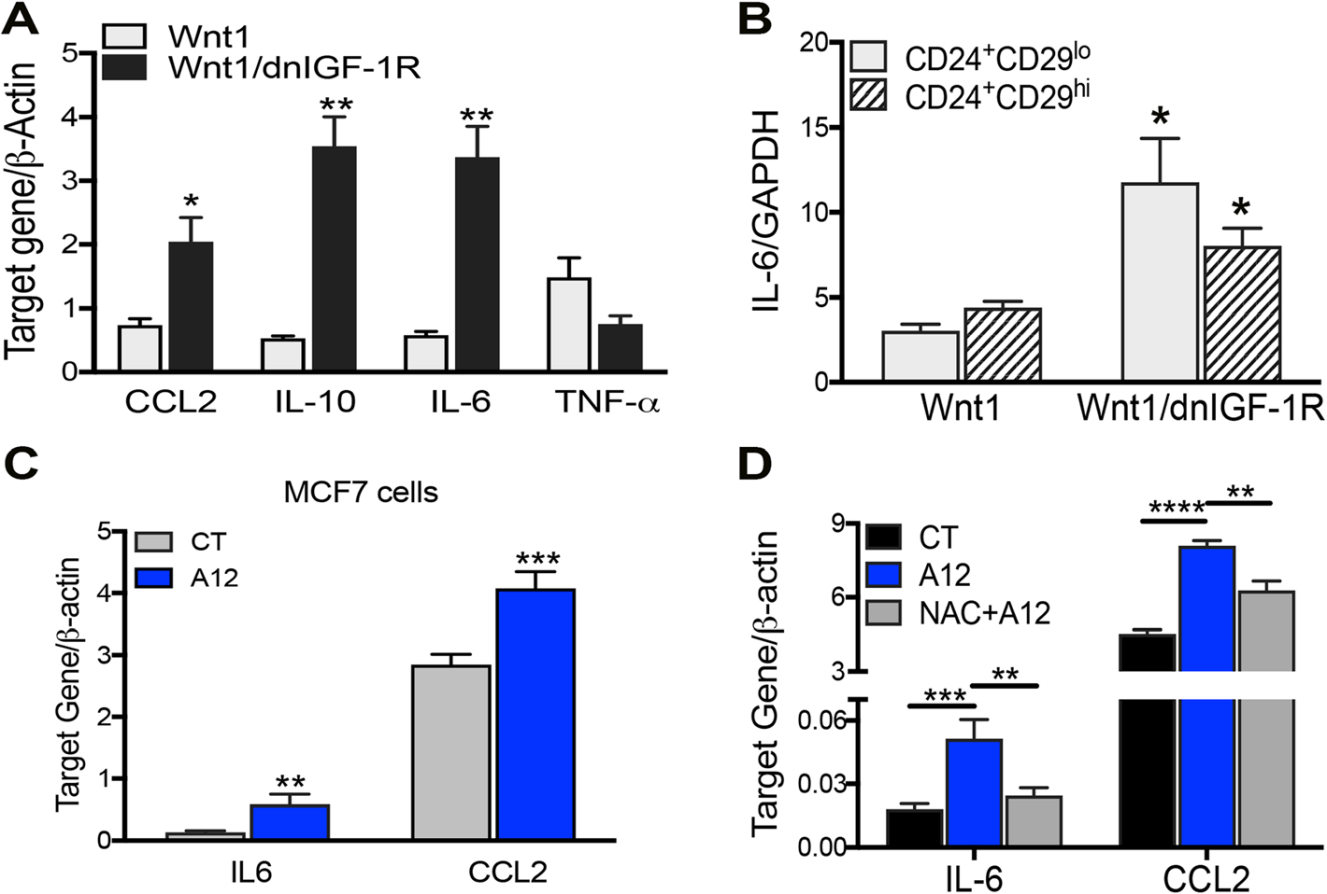
Reduced insulin-like growth factor type1 receptor (IGF-1R) alters cytokine and chemokine production in tumor epithelia. **a** RT-PCR analysis of CCL2, IL-10, IL-6, and TNF-α in *Wnt1/dnIGF-1R* versus *Wnt1* tumors (Student’s *t* test *P < 0.05, **P < 0.01; *n* = 5). **b** RT-PCR analysis of IL-6 in CD24^+^CD29^lo^, Lin^−^ (CD45, CD31, Gr-1, Ter-119) luminal cells and CD24^+^CD29^hi^, Lin^−^ (CD45, CD31, Gr-1, Ter-119) basal cells from *Wnt1/dnIGF-1R* versus *Wnt1* tumors (Student’s *t* test *P < 0.05; *n* = 4, 3 technical replicates per sample). **c** RT-PCR analysis of IL-6 and CCL2 in human IgG (IgG) or IMC-A12 (A12) treated MCF7 cells (Student’s *t* test **P < 0.01, ***P < 0.001; *n* = 3, with three biological replicates per experiment). **d** RT-PCR analysis of IL-6 and CCL2 in MCF7 cells treated with IgG, A12, or A12 + N-acetyl-L-cysteine (NAC) (one-way analysis of variance compared to A12, **P < 0.01, ***P < 0.001, ****P < 0.0001; *n* = 3, with three biological replicates per experiment)

Activation of cellular stress results in an epithelial inflammatory response by production of cytokines and chemokines [38, 39]. Thus, we measured cytokine and chemokine gene expression in our mouse tumors with reduced IGF-1R activity by employing a targeted qRT-PCR array specifically designed for known altered cytokines and chemokines in solid tumors [22]. By measuring expression in the whole tumor, we found alterations in 20 cytokines and chemokines in *MMTV-Wnt1/dnIGF-1R* compared to *MMTV-Wnt1* tumors (Additional file 4: Table S3). Specifically, we observed an increase in C-C motif chemokine ligand 2 (CCL2), interleukin-10 (IL-10), and interleukin-6 (IL-6) whereas tumor necrosis factor-alpha (TNF-α) was decreased in *MMTV-Wnt1/dnIGF-1R* tumors (Fig. 3a). Alterations in these cytokines/chemokines suggest a tumor-promoting immune cell microenvironment [40, 41].

Previous studies have shown IL-6 is expressed in tumor epithelial cells resulting in tumor cell growth [42]. We investigated whether the loss of IGF-1R function altered IL-6 expression in the tumor epithelial cell population by measuring IL-6 expression in the CD24^+^/CD29^lo^ (luminal) and CD24^+^/CD29^hi^ (basal) cell populations. Interestingly, IL-6 messenger RNA (mRNA) expression was significantly increased in both CD24^+^/CD29^lo^ and CD24^+^/CD29^hi^ epithelial cells (Fig. 3b). Furthermore, previous studies have shown both IL-6 and CCL2 are expressed in human breast cancer cell lines and enhance tumor growth, migration, and immune cell recruitment [43, 44]. MCF7 cells with IGF-1R inhibition had increased IL-6 and CCL2 gene expression (Fig. 3c) and IL-6 protein expression (Additional file 5: Figure S2), further supporting the conclusion that attenuation of IGF-1R increases epithelial specific cytokine and chemokine expression.

We further tested whether increased IL-6 and CCL2 expression in tumor epithelial cells is a direct result of increased cellular stress in response to IGF-1R inhibition. To demonstrate that increased cellular stress activated through the loss of IGF-1R directly results in altered IL-6 and CCL2 expression, we blocked ROS production using a ROS scavenger, N-acetyl-L-cysteine (NAC) in MCF7 cells treated with the IGF-1R blocking antibody. Scavenging of ROS by NAC resulted in decreased IL-6 and CCL2 gene expression (Fig. 3d) suggesting direct regulation of cytokine production through cellular stress activation driven by attenuated IGF-1R function.

### Attenuated IGF-1R enhances tumor immune cell invasion

**Fig. 4:**
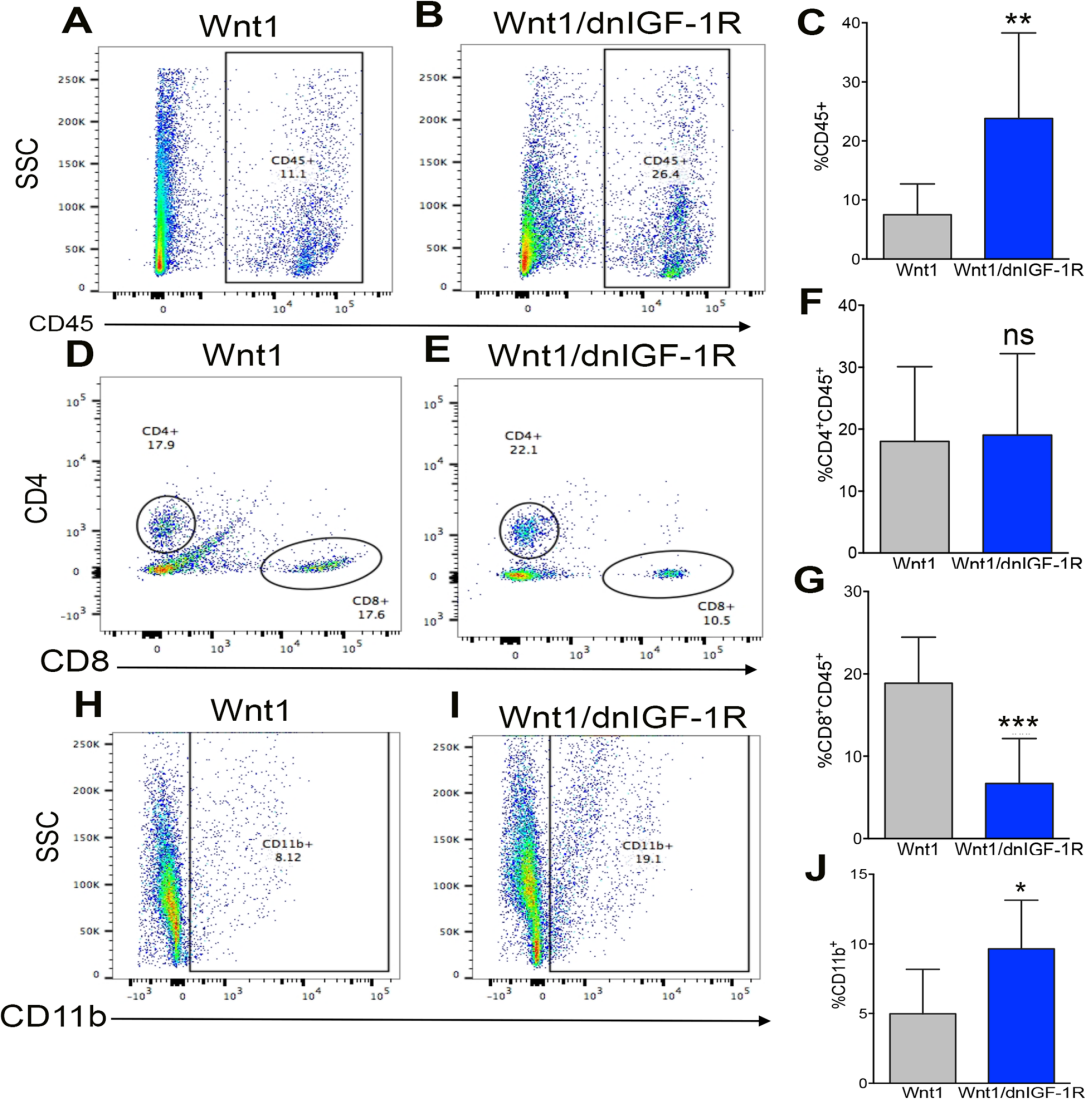
*MMTV-Wnt1/dnIGF-1R* tumors have altered immune cell infiltration. **a**, **b** Representative dot plot derived from flow cytometry measuring leukocyte marker CD45-PE/Cy5 of purified immune cells from *Wnt1* (**a**) and *Wnt1/dnIGF-1R* (**b**) tumors. **c** Quantification of flow cytometry of CD45^+^ leukocytes in *Wnt1* versus *Wnt1/dnIGF-1R* tumors (*Wnt1/dnIGF-1R* versus *Wnt1* **P < 0.01; *n* = 20 each group). **d**, **e** representative dot plot of flow cytometry using cell surface markers to select for helper/regulatory T cells (CD4^+^CD45^+^) or cytotoxic T cells (CD8^+^CD45^+^) in *Wnt1* (**d**) or *Wnt1/dnIGF-1R* (**e**) tumors. **f, g** Quantification of flow cytometry of CD4^+^CD45^+^-stained T cells (**f**) or CD8^+^CD45^+^-stained cytotoxic T cells (**g**) in *Wnt1* versus *Wnt1/dnIGF-1R* tumors (CD4^+^CD45^+^ *Wnt1/dnIGF-1R* cells versus *Wnt1* cells *P* > 0.05 (ns, not significant); CD8^+^CD45^+^ *Wnt1/dnIGF-1R* cells versus *Wnt1* cells ***P < 0.001; *n* = 20 each group). **h**, **i** representative dot plot of flow cytometry using monocyte marker CD11b-PerCP of purified immune cells from *Wnt1* (**h**) and *Wnt1/dnIGF-1R* (**i**) tumors. **j** Quantification of flow cytometry of CD11b^+^ monocytes in *Wnt1* versus *Wnt1/dnIGF-1R* tumors (*Wnt1/dnIGF-1R* versus *Wnt1* *P < 0.05; *n* = 20 each group)

Alterations in cytokine and chemokine production in the tumor epithelium as a result of attenuated IGF-1R signaling suggests changes in immune cell recruitment to the primary tumor. The immune microenvironment is a major component of the primary tumor critical for maintaining either a tumorigenic or tumor cytotoxic environment dependent on the primary tumor immune cell profile and cytokine/chemokine production [45, 46]. Immune cell profiling by flow cytometry revealed an increase in total leukocytes stained for CD45 (Fig. 4a, b) in *MMTV-Wnt1/dnIGF-1R* compared to *MMTV-Wnt1* tumors. Surprisingly, helper/regulatory CD4^+^ T cells (CD4^+^, CD45^+^), known to promote tumor growth, were unchanged (Fig. 4c, d), and additional flow cytometry analysis by CD25 and FOXP3 staining showed no change in regulatory T cells (Additional file 6: Figure S3). Cytotoxic T cells (CD8^+^, CD45^+^), however, were significantly decreased in *MMTV-Wnt1/dnIGF-1R* tumors (Fig. 4e, f) suggesting a reduced tumor cytotoxic immune microenvironment. We also observed an increase in CD11b^+^, CD45^+^ monocytes (Fig. 4g, h) in *MMTV-Wnt1/dnIGF-1R* tumors indicating an influx of immune cells with potential to differentiate into tumor-associated macrophages (TAMs). Taken together, these data indicate that reduced epithelial IGF-1R function in *MMTV-Wnt1* tumors results in an influx of immune cells that enhance tumor growth and contribute to an aggressive tumor microenvironment.

### Production of IL-6/CCL2 due to attenuation of IGF-1R promotes tumor monocyte migration

**Fig. 5:**
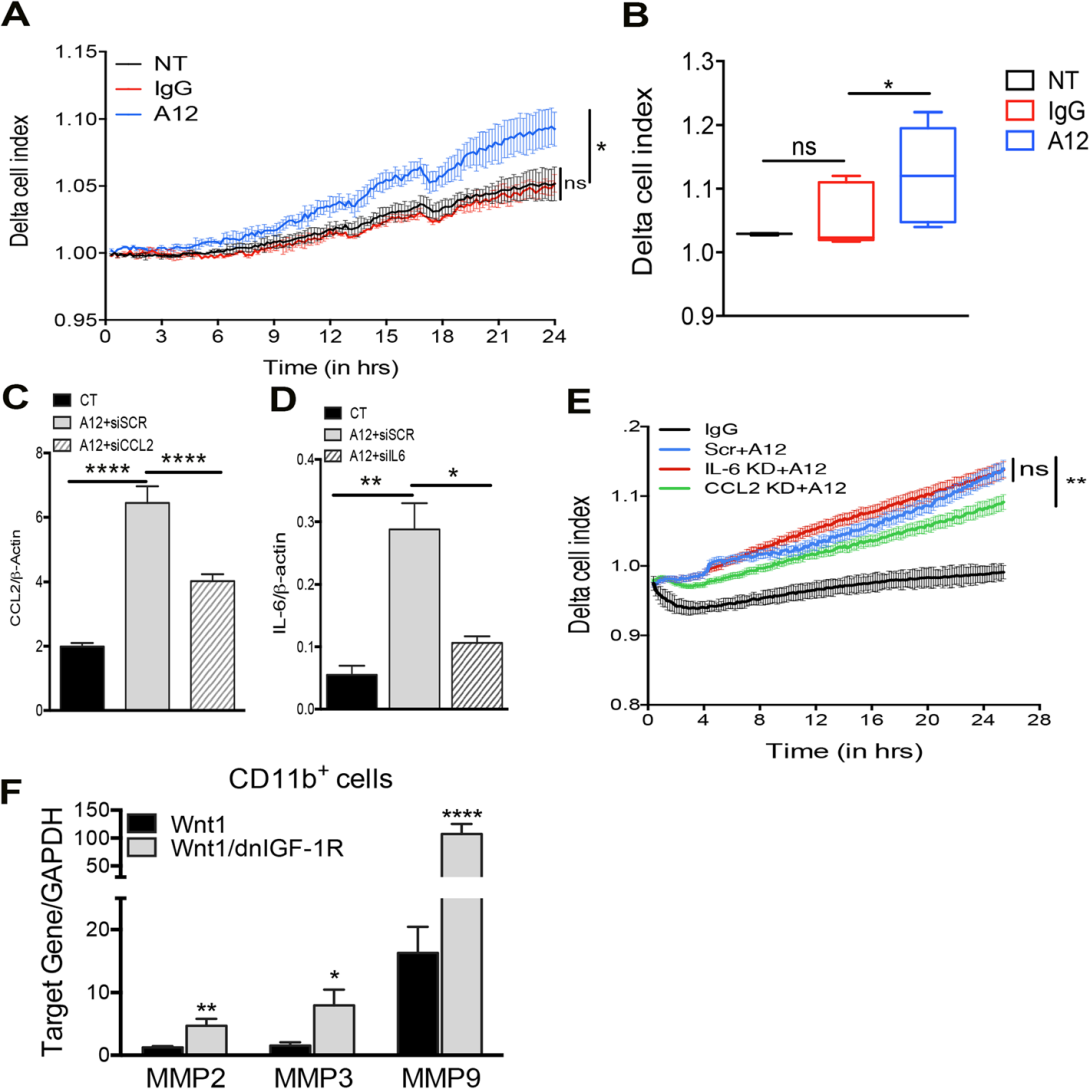
Knockdown of C-C motif chemokine ligand 2 (CCL2) decreases monocyte migration towards A12-treated MCF7 cells. **a**, **b** Representative chart of RAW264.7 monocyte migration depicted as delta cell index over time (in hours) towards untreated (NT; black), human IgG-treated (IgG; red), or IMC-A12-treated (A12; blue) MCF7 cells (**a**) and quantification at 24 h (**b**) (one-way analysis of variance (ANOVA): *P < 0.05; *n* = 3, with four technical replicates in each experiment). **c**, **d** RT-PCR analysis of CCL2 (**c**) and IL-6 (**d**) in IgG-treated (CT) or A12-treated MCF7 cells after transfection with non-targeting scramble (Scr) or target-specific small interfering RNA (siRNA) (siIL-6, siCCL2) (one-way ANOVA: *P < 0.05, **P < 0.01, ****P < 0.0001; *n* = 3, with three biological replicates per experiment). **e** chart of RAW264.7 monocyte migration depicted as delta cell index over time (in hours) towards human IgG-treated (IgG; black), siScramble + A12-treated (Scr + A12; blue), siIL-6 + A12-treated (IL-6 KD + A12, red), or siCCL2 + A12-treated (CCL2 KD + A12, green) MCF7 cells (one-way ANOVA; *n* = 3, with four technical replicates in each experiment). **f** RT-PCR analysis of matrix metalloproteinase (MMP)-2, MMP-3, and MMP-9 in *Wnt1/dnIGF-1R* versus *Wnt1* CD11b^+^ monocytes (Student’s *t* test *P < 0.05, **P < 0.01, ****P < 0.0001; *n* = 4, with three technical replicates per sample). GAPDH, glyceraldehyde-3-phosphate dehydrogenase

To test whether attenuated IGF-1R function in human breast tumor cells directly alters the recruitment of monocytes, we modeled migration in vitro with the xCELLigence RTCA DP real-time migration assay using the MCF7 breast cancer cell line. RAW264.7 monocyte migration increased towards MCF7 cells treated with A12, compared to the control-untreated or IgG-antibody-treated MCF7 cells (Fig. 5a, b). Prior studies demonstrated production of CCL2 and IL-6 in breast epithelial cells is responsible for monocyte recruitment to solid tumors [47, 48]. To test whether CCL2 or IL-6 production due to attenuated IGF-1R function directly alters the recruitment of monocytes, we again modeled migration in vitro. RAW264.7 monocyte migration increased towards MCF7 cells with IGF-1R inhibition (Fig. 5a-b, e) which was decreased with CCL2 knockdown (Fig. 5d, e), but not with IL-6 knockdown (Fig. 5c, e) suggesting production of CCL2 through IGF-1R inhibition enhances monocyte migration.

Previously, it was shown that TAMs produce matrix metalloproteinases (MMPs) to degrade tumor matrix and promote tumor cell extravasation [49]. To test for MMP production in the monocyte specific population of *MMTV-Wnt1/dnIGF-1R* tumors we analyzed *MMP* gene expression in isolated CD11b^+^ monocytes. *MMP-2*, *MMP-3*, and *MMP-9* levels were significantly increased in CD11b^+^ monocytes from *MMTV-Wnt1/dnIGF-1R* tumors (Fig. 5f). These data suggest the increased accumulation of monocytes in *MMTV-Wnt1/dnIGF-1R* tumors promotes tumorigenesis through production of MMPs [49].

### Reduced IGF-1R signaling in Wnt1-driven mammary tumors promotes an aggressive tumor microenvironment

**Fig. 6:**
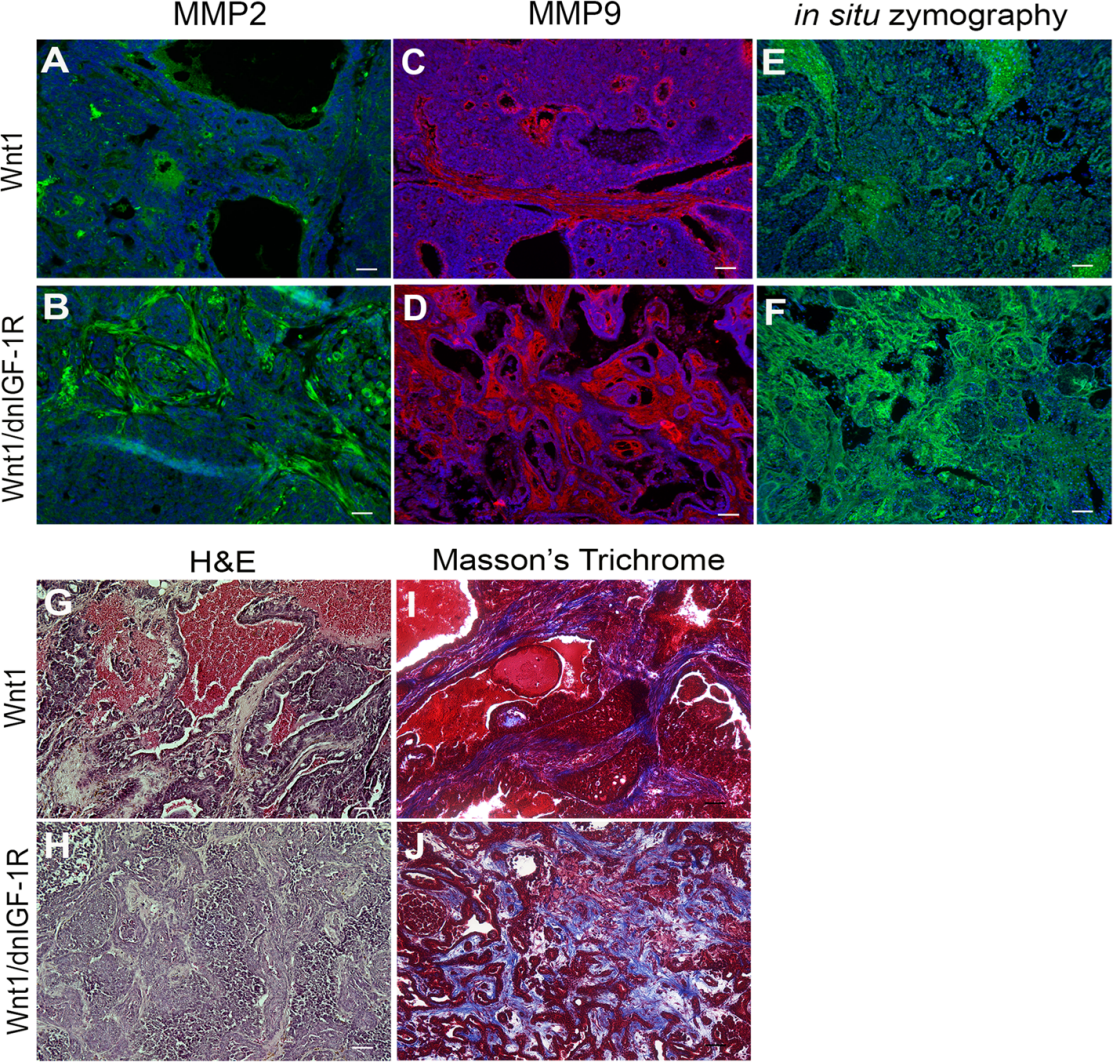
Increased aggressive tumor microenvironment in *MMTV-Wnt1/dnIGF-1R* compared to *MMTV-Wnt1* tumors. **a**-**d** Representative photomicrographs showing immunofluorescence staining of matrix metalloproteinase (MMP)2 (green) and MMP9 (red) in *Wnt1* (**a**, **c**) and *Wnt1/dnIGF-1R* (**b**, **d**) tumor sections. **e**, **f** Representative photomicrographs depicting active MMPs through *in situ* zymography (green) in *Wnt1* (**e**) and *Wnt1/dnIGF-1R* (**f**). **g**, **h** Representative H&E-stained tissue sections from *MMTV-Wnt1 (Wnt1)* (**g**) and *MMTV-Wnt1/dnIGF-1R (Wnt1/dnIGF-1R)* (**h**) tumors. **i**, **j** Representative Masson’s Trichrome-stained tissue sections for collagen (blue) in *Wnt1* (**i**) and *Wnt1/dnIGF-1R* (**j**). Sections (**a**-**f**) were stained with 4',6-diamidino-2-phenylindole to detect nuclei (blue). Scale bar = 100 μm

Breakdown of the tumor basement membrane and surrounding matrix is critical for tumor cell extravasation and restructuring of the tumor microenvironment. MMPs are overexpressed and actively secreted in primary tumors that maintain an aggressive tumor microenvironment [50]. The accumulation of monocytes in *MMTV-Wnt1/dnIGF-1R* tumors that produce MMPs suggests potential for secretory activity and matrix remodeling. Immunostaining revealed increased MMP2 and MMP9 in the stromal compartment of *MMTV-Wnt1/dnIGF-1R* tumors (Fig. 6a-d). Using in situ zymography, we further determined that the increased MMP expression correlated with increased MMP activity within the stroma of tumors with reduced IGF-1R signaling (Fig. 6e, f).

Increased MMP secretion and activity in tumors with attenuated IGF-1R suggests there is active tumor stroma matrix remodeling. With hematoxylin and eosin staining, we observed morphological alterations in the tumor stroma with attenuated IGF signaling (*MMTV-Wnt1/dnIGF-1R)* (Fig. 6g, h). Further analysis by Masson’s Trichrome staining revealed increased collagen in tumors with IGF-1R inhibition (Fig. 6i, j), which is associated with increased risk of metastasis [51]. These data suggest the primary tumor actively remodels the matrix in response to reduced IGF-1R function in the tumor epithelium.

### Low IGF-1R expression inversely correlates with cytokine and MMP expression in humans

**Fig. 7:**
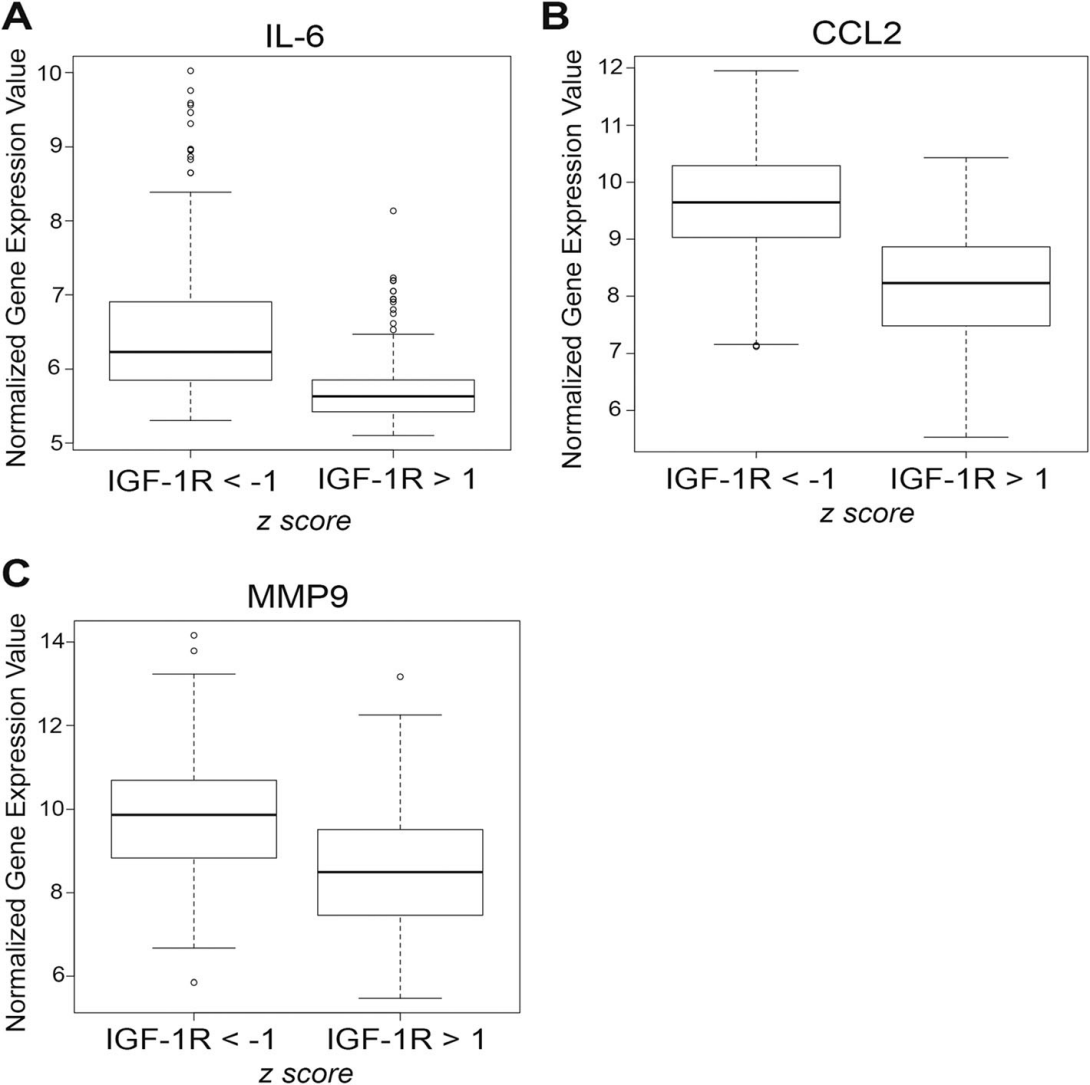
Insulin-like growth factor type1 receptor (IGF-1R) expression is inversely correlated with cytokine and matrix metalloproteinase (MMP) expression in human breast cancer. **a**-**c** Analysis of gene expression from the Molecular Taxonomy of Breast Cancer International Consortium (METABRIC) dataset in human breast tumors with low (IGF-1R z-score < − 1) versus high (IGF-1R z-score > 1) IGF-1R expression determined by z-score. Representation of IL-6 (**a**) (Student’s *t* test; *P* < 2.2 × 10^− 16^), C-C motif chemokine ligand 2 (CCL2) (**b**) (Student’s *t* test; *P* < 2.2 × 10^− 16^), and MMP9 (**c**) (Student’s *t* test; *P* < 2.2 × 10^− 16^) expression

Previous analysis of the human patient gene expression datasets revealed IL-6, CCL2, and MMP9 expression are upregulated in TNBC compared to ER+/PR+ breast cancer [42, 48]. Our analysis of the METABRIC dataset confirmed increased expression of IL-6, CCL2, and MMP9 in TNBC (Additional file 7: Figure S4). Further analysis revealed IL-6, CCL2, and MMP9 were upregulated in breast tumors with low compared to high IGF-1R (Fig. 7a-c). Moreover, CHOP and MMP2 expression were upregulated in tumors with low IGF-1R, whereas expression was unchanged in TNBC compared to ER+/PR+ breast cancer (Additional file 7: Figure S4) revealing CHOP and MMP2 are more correlated with IGF-1R levels than with hormone receptor status. Taken together, we determined that IL-6, CCL2, MMP9, MMP2, and CHOP expression are all inversely correlated with IGF-1R expression in human breast cancer.

## Discussion

Historically, IGF-1R was proposed to have oncogenic effects in breast tumorigenesis, where overexpression or hyperactivation leads to increased tumor cell proliferation and survival [6, 9, 10]. However, recent reports have suggested that overexpression of IGF-1R in ER+/PR+ breast cancers results in a favorable prognosis [6, 15], and low expression of IGF-1R leads to a more undifferentiated tumor phenotype and worse overall survival [8, 13] (Fig. 1). Consistent with these reports, we previously demonstrated that expression of a dominant-negative IGF-1R in a basal-like breast cancer tumor mouse model driven by the Wnt1 oncogene resulted in a more undifferentiated tumor and metastatic phenotype [1]. Interestingly, inhibitors of IGF-1R have been unsuccessful in the clinic, and the cause for this is still not understood [11, 12]. The exacerbated phenotype in the dominant-negative IGF-1R tumors raises the question of whether IGF-1R and the Wnt pathway interact in breast tumorigenesis. METABRIC analysis of several Wnt signaling targets overexpressed in breast cancer revealed that Wnt2 and Frizzled 9 (Fzd9) were inversely correlated with IGF-1R expression in humans (Additional file 8: Figure S5). Interestingly, unbiased analysis with a targeted Wnt signaling array identified Wnt2 and Fzd9 as upregulated (9.3-fold, 2.3-fold) in *MMTV-Wnt1/dnIGF-1R* compared to *MMTV-Wnt1* tumors (Additional file 8: Figure S5), further linking the bigenic mouse model with human disease. Previous studies have shown upregulated Wnt2 in the primary breast tumor is linked to metastatic disease [52], and more recently, Wnt2 was identified as a regulator of tumor initiation in a basal-like breast cancer model [53]. Moreover, Wnt2 is the only Wnt ligand that activates Fzd9, subsequently activating the canonical Wnt signaling pathway [54].

The mechanisms by which decreased IGF-1R function might contribute to an aggressive, invasive tumor phenotype have not been identified. In this study, we revealed a novel role for IGF-1R as a suppressor of tumorigenesis by regulating the tumor microenvironment through protecting tumor epithelial cells from EnR stress. Previously, it was found that a reduction in IGF signaling increased cellular stress and production of ROS in vascular smooth muscle cells, 3T3-L1 adipocytes, and MCF7 cells [21, 55–57]. Moreover, IGF-1 stimulation protects against thapsagargin-induced EnR stress activity in MCF7 cells suggesting the IGF-1 signaling pathway augments the adaptability of breast tumor cells to EnR stress [19]. Increased oxidative stress results in production of cytokines in tumor epithelium [38, 58] and a pro-inflammatory response in a number of tissues [59]. In our study, cellular oxidative stress was increased as a result of attenuating IGF-1R directly resulting in cytokine production, specifically IL-6 and CCL2 (Figs. 2, 3). Previous studies have also shown increased production of ROS activates the EnR stress pathway; this subsequent activation increases the production of ROS through PDI resulting in a positive feedback loop [16, 17]. We showed that inhibition of IGF-1R in tumor epithelium results in activation of the EnR stress pathway and upregulation of PDI suggesting that the increased ROS production may be through amplified EnR stress.

Both IL-6 and CCL2 play a major role in promoting tumorigenesis by altering the primary tumor immune microenvironment and enhancing a more aggressive primary tumor phenotype [43, 44, 60]. Importantly, IL-6 and CCL2 production in mouse and human tumor epithelial cells is elevated with reduced IGF-1R function (Fig. 3). As a result of altered cytokine production within the primary tumor epithelium, we observed altered immune cell invasion in *MMTV-Wnt1/dnIGF-1R* tumors. The decrease in CD8^+^ cytotoxic T cells indicates fewer immune cells responsible for tumor degradation present within the primary tumors lacking IGF-1R function. Furthermore, we observed an increased influx of CD11b^+^ monocytes that have potential to polarize into tumor-associated macrophages (TAMs) dependent on surrounding signals from the tumor (Fig. 4). Moreover, consistent with previous reports we showed that CCL2 is necessary for macrophage migration [47]. Although IL-6 is not necessary for macrophage recruitment in our model, the upregulation of IL-6 in tumor epithelium may be important for other functions such as tumor initiation, growth, and metastasis [42, 43, 61] (Fig. 5). Although monocyte recruitment is increased in tumors with attenuated IGF-1R signaling, it is still unclear whether these monocytes become tumor-degrading or tumor-promoting macrophages. TAMs involved in tumorigenesis are known to produce matrix metalloproteinases (MMPs) [49]. The increased production of MMPs within the monocytes from *MMTV-Wnt1/dnIGF-1R* tumors suggests an enrichment of tumor-promoting TAMs (Fig. 5). Therefore, the composition of the immune microenvironment in *MMTV-Wnt1/dnIGF-1R* primary tumors favors tumor growth and extravasation.

Prior studies have shown that increased expression of key tumor microenvironment components such as collagen and MMP2/MMP9 are necessary for tumor epithelial extravasation [50, 51, 62, 63]. Secretion of active MMP2 and MMP9 into the stroma of primary tumors allows for break-down of the surrounding tumor matrix and matrix remodeling to promote epithelial cell invasion [64]. Here, we observed increased MMP activity within the stroma of *MMTV-Wnt1/dnIGF-1R* tumors that corresponded with the increased MMP2 and MMP9 expression (Fig. 6). De novo collagen deposition is necessary during tumor matrix remodeling and increased collagen is correlated with metastatic tumors [65, 66]. Increased collagen levels measured by Masson’s Trichrome staining revealed that tumors lacking a functional IGF-1R have a significantly altered tumor microenvironment that is consistent with promoting tumor cell invasion (Fig. 6).

It is well-known that IL-6, CCL2, and MMP9 expression are increased in TNBC compared to ER+/PR+ breast cancer [42, 48, 67]. Consistent with our mouse tumor model with reduced IGF-1R signaling, low IGF-1R is inversely correlated with IL-6, CCL2, and MMP9 expression in human tumors (Fig. 7). Interestingly, MMP2 and CHOP were also upregulated in human tumors with low IGF-1R, similar to our mouse model, whereas these two genes were unchanged in TNBC compared to ER+/PR+ breast cancer. Taken together, our data suggest this set of genes is specifically correlated with human tumors that have low levels of IGF-1R.

## Conclusions

**Fig. 8:**
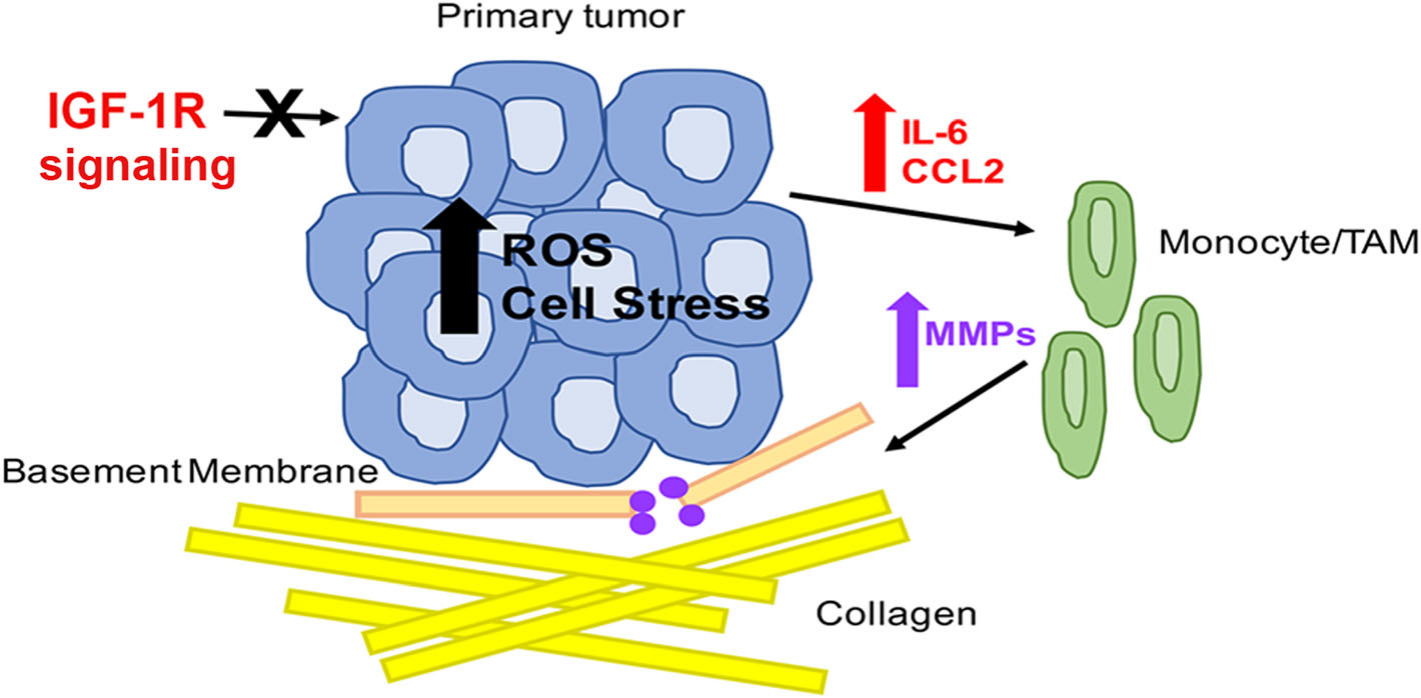
Model for insulin-like growth factortype 1 receptor (IGF-1R) regulation of primary tumor cellular stress and enhancement of an aggressive tumor microenvironment. Reduced or inhibited IGF-1R function results in increased reactive oxygen species (ROS) accumulation and primary tumor epithelial endoplasmic reticulum (EnR) stress leading to increased production of IL-6 and C-C motif chemokine ligand 2 (CCL2) by the primary tumor epithelium. Secretion of IL-6 and CCL2 signals for monocyte infiltration and differentiation to tumor-associated macrophages (TAM). This differentiation increases MMP expression and secretion resulting in active tumor basement membrane breakdown allowing for increased collagen deposition to provide an environment for tumor cell extravasation

We have defined IGF-1R as a negative regulator of cytokine production by protecting epithelial cells from oxidative stress, resulting in maintenance of a tumor microenvironment that suppresses tumor cell invasion (Fig. 8). Taken together, these data support a protective function of the IGF-1R in breast epithelial cells and suggest that reduction of IGF-1R signaling in the epithelial cells leads to increased ROS production and EnR stress, altered cytokine production, and as a result, tumor microenvironment remodeling to promote cellular invasion and metastasis.

## Additional files

Additional file 1: **Table S1.** *MMTV-Wnt1* tumor phenotype is altered with reduced IGF-1R [1]. (DOCX 16 kb)

Additional file 2: **Table S2.** qRT-PCR primer list. (DOCX 14 kb)

Additional file 3: **Figure S1.** IMC-A12 blocks activation of IGF-1R signaling. Western blot analysis of pAkt (473) and pIGF-1R in IgG or A12 treated MCF7 cells with or without IGF-1. (TIF 339 kb)

Additional file 4: **Table S3.** Cytokine and chemokine profile is altered in *MMTV-Wnt1* tumors with attenuated IGF-1R. Gene expression fold change measured by the ∆∆Ct method comparing *MMTV-Wnt1 and MMTV-Wnt1/dnIGF-1R tumors.* Student’s *t* test was performed to determine the corresponding *p* values. (DOCX 25 kb)

Additional file 5: **Figure S2.** IL-6 protein expression is increased in tumor cells with reduced IGF signaling. ELISA analysis of IL-6 in IgG or A12 treated MCF7 cells (Student’*s t* test *P < 0.05, *n* = 3; 3 biological replicates per experiment). (TIF 57 kb)

Additional file 6: **Figure S3.** Reduced IGF-1R in primary tumors does not alter the active T cell population. (A, B) Quantification of flow cytometry of CD3^+^ T cells (A) and activated regulatory T cells positive for FOXP3 and CD25 (B) in *Wnt1* versus *Wnt1/dnIGF-1R* tumors (*Wnt1/dnIGF-1R* versus *Wnt1* **P < 0.01; *n* = 20 each group). (TIF 70 kb)

Additional file 7: **Figure S4.** Cytokine and MMP expression in human breast cancer patients. Analysis of gene expression from the METABRIC dataset. (A-C) Boxplot representation of IL-6 (A) (Student’s *t* test, *P* < 2.2 × 10^− 16^), CCL2 (B) (Student’s *t* test, *P* < 2.2 × 10^− 16^), and MMP9 (C) (Student’s *t* test, *P* < 2.2 × 10^− 16^) expression levels in ER+/PR+ breast cancer compared to triple-negative breast cancer (TNBC). (D-G). Boxplot representation of MMP2 and CHOP (D, F) expression levels in ER+/PR+ breast cancer compared to TNBC (MMP2: Student’s *t* test, *P* < 2.2 × 10^− 16^; CHOP: *P* < 2.2 × 10^− 16^). Boxplot representation of MMP2 and CHOP (E, G) expression in human breast tumors with low (IGF-1R z-score < − 1) versus high (IGF-1R z-score > 1) IGF-1R expression (Student’s *t* test, MMP2, *P* < 6.864 × 10^− 10^; CHOP, *P* < 3.172 × 10^− 10^). (TIF 253 kb)

Additional file 8: **Figure S5.** Wnt2 and Frizzled9 expression are inversely correlated with IGF-1R expression in breast cancer. (A, B) Analysis of gene expression from the METABRIC dataset. Boxplot representation of Wnt2 (A) (Student’s *t* test, *P* < 2.2 × 10^− 16^) and Fzd9 (Frizzled9) (B) (Student’s *t* test, *P* < 2.2 × 10^− 16^) in human breast tumors with low (IGF-1R z-score < − 1) versus high (IGF-1R z score > 1) IGF-1R expression. (C, D). qRT-PCR analysis of Wnt2 (C) and Fzd9 (D) in *MMTV-Wnt1* compared to *MMTV-Wnt1/dnIGF-1R* tumors (*n* = 4). (TIF 232 kb)

## Abbreviations

ANOVA: Analysis of variance
ATCC: American Type Culture Collection
BSA: Bovine serum albumin
CCL2: C-C motif chemokine ligand 2
CHOP: C/EBP homologous protein
DAB: 3,3′-Diaminobenzidine
DCFDA: 2′,7′-Dichlorofluorescin diacetate
DMEM: Dulbecco’s modified Eagle’s medium
eIF2-α: Eukaryotic initiation factor 2-alpha
ELISA: Enzyme-linked immunosorbent assay
EnR: Endoplasmic reticulum
ER+/PR+: Estrogen receptor/progesterone receptor-positive
FACS: Fluorescence-activated cell sorting
FBS: Fetal bovine serum
GAPDH: Glyceraldehyde-3-phosphate dehydrogenase
HR: Hormone receptor
IGF-1: Insulin-like growth factor-1
IGF-1R: Insulin-like growth factortype 1 receptor
IHC: Immunohistochemistry
IL-10: Interleukin-10
IL-6: Interleukin-6
LumA: Luminal A
LumB: Luminal B
MEC: Mammary tumor epithelial cell
MEM: Minimum essential medium
METABRIC: Molecular Taxonomy of Breast Cancer International Consortium
MMP: Matrix metalloproteinase
NAC: N-acetyl cysteine
PAM50: Prediction analysis of microarray 50
PBS: Phosphate-buffered saline
PDI: Protein disulfide isomerase
qRT-PCR: Quantitative real-time polymerase chain reaction
RIPA: Radioimmunoprecipitation assay
ROS: Reactive oxygen species
Scr: Scramble
siRNA: Small interfering RNA
TAM: Tumor-associated macrophage
TCGA: The Cancer Genome Atlas
TNBC: Triple-negative breast cancer
TNF-α: Tumor necrosis factor-alpha
UPR: Unfolded protein response
