# Supplementary material for: Insulin-like growth factor receptor signaling in breast tumor epithelium protects cells from endoplasmic reticulum stress and regulates the tumor microenvironment
Source: Breast Cancer Res. 2018 Nov 20;20:138. doi: 10.1186/s13058-018-1063-2 (PMC6245538; doi:10.1186/s13058-018-1063-2)
Supplement: Supplementary file 1 — Table S1. MMTV-Wnt1 tumor phenotype is altered with reduced IGF-1R [1]. (DOCX 16 kb) [file 13058_2018_1063_MOESM1_ESM.docx]

|  | *MMTV-Wnt1/dnIGF-1R compared to MMTV-Wnt1* |
| --- | --- |
| Tumor latency | Decreased 100 days |
| Tumor growth | No change |
| Basal phenotype | Increased CD24^+^CD29^hi^ basal cell population by 15% |
| Pulmonary metastasis | Increased 100% |

**Table S1.** *MMTV-Wnt1* tumor phenotype is altered with reduced IGF-1R [[1](#_ENREF_1)].
