# Supplementary material for: Insulin-like growth factor receptor signaling in breast tumor epithelium protects cells from endoplasmic reticulum stress and regulates the tumor microenvironment
Source: Breast Cancer Res. 2018 Nov 20;20:138. doi: 10.1186/s13058-018-1063-2 (PMC6245538; doi:10.1186/s13058-018-1063-2)
Supplement: Supplementary file 2 — Table S2. qRT-PCR primer list. (DOCX 14 kb) [file 13058_2018_1063_MOESM2_ESM.docx]

| Gene Target | Forward Primer 5’ to 3’ | Reverse Primer 5’ to 3’ |
| --- | --- | --- |
| Mouse CCL2 | CATCCACGTGTTGGCTCA | GATCATCTTGCTGGTGAATGAGT |
| Mouse Fzd9 | TTTCTTCTCCACGGCCTTC | GGTACTGGAACCGGTGAGG |
| Mouse GAPDH | GATGCCCCCATGTTTGTGAT | GGTCATGAGCCCTTCCACAAT |
| Mouse IL-6 | AGCCAGCATTGTGGGTTG | CGGCAAGTGAGCAGATAGC |
| Mouse IL-10 | CAGCCGGGAAGACAATAACT | GTTGTCCAGCTGGTCCTTTG |
| Mouse MMP2 | GCATCTACTTGCTGGACATCAG | AGGATACCCCAAGCCACTG |
| Mouse MMP3 | AGCTGAGGACTTTCCAGGTG | TGCGAAGATCCACTGAAGAA |
| Mouse MMP9 | ACGACATAGACGGCATCCA | GCTGTGGTTCAGTTGTGGTG |
| Mouse TNFa | CTGTAGCCCACGTCGTAGC | TTGAGATCCATGCCGTTG |
| Mouse Wnt2 | GGTCAGCTCTTCATGGTGGT | TGGCACATTGTCACACATCA |
| Human Beta-Actin | AGCCATGTACGTTGCTATCCA | ACCGGAGTCCATCACGATG |
| Human CCL2 | AGTCTCTGCCGCCCTTCT | GTGACTGGGGCATTGATTG |
| Human IL-6 | CAGGAGCCCAGCTATGGACT | GAAGGCAGCAGGCAACAC |
