# Supplementary figures and images for: Insulin-like growth factor receptor signaling in breast tumor epithelium protects cells from endoplasmic reticulum stress and regulates the tumor microenvironment
Source: Breast Cancer Res. 2018 Nov 20;20:138. doi: 10.1186/s13058-018-1063-2 (PMC6245538; doi:10.1186/s13058-018-1063-2)

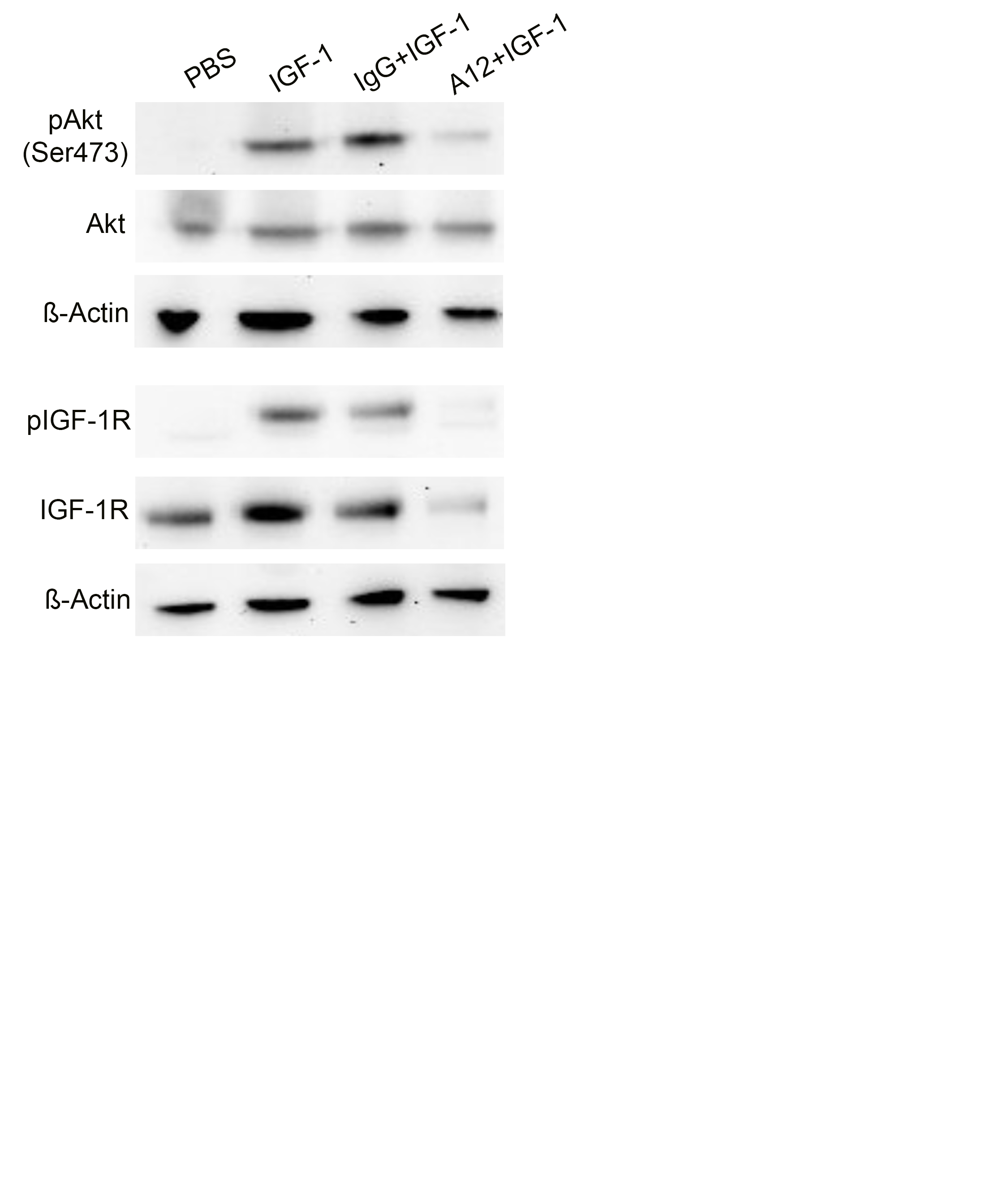

Supplement: Supplementary file 3 — Figure S1. IMC-A12 blocks activation of IGF-1R signaling. Western blot analysis of pAkt (473) and pIGF-1R in IgG or A12 treated MCF7 cells with or without IGF-1. (TIF 339 kb) [file 13058_2018_1063_MOESM3_ESM.tif]

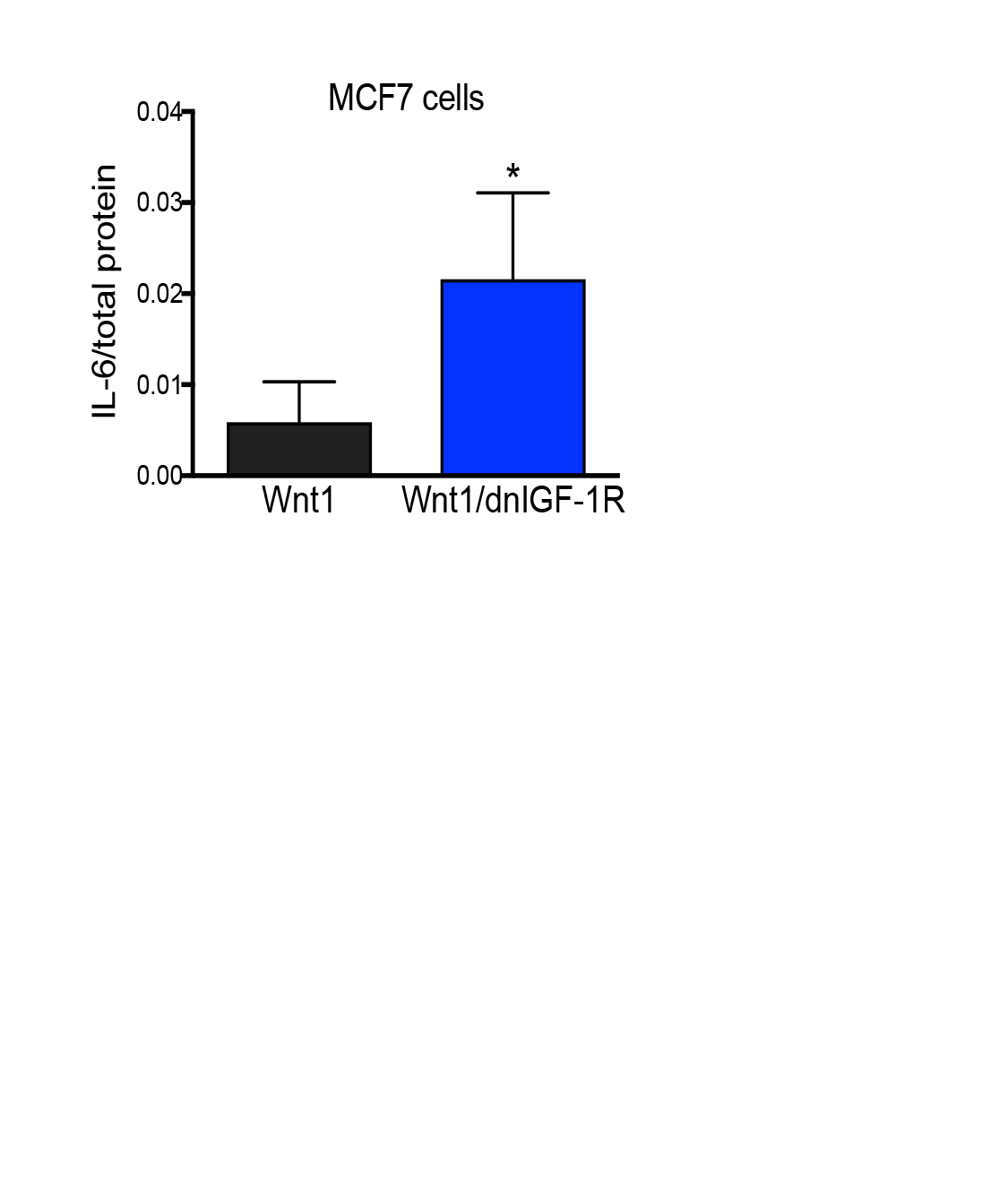

Supplement: Supplementary file 5 — Figure S2. IL-6 protein expression is increased in tumor cells with reduced IGF signaling. ELISA analysis of IL-6 in IgG or A12 treated MCF7 cells (Student’s t test *P < 0.05, n = 3; 3 biological replicates per experiment). (TIF 57 kb) [file 13058_2018_1063_MOESM5_ESM.tif]

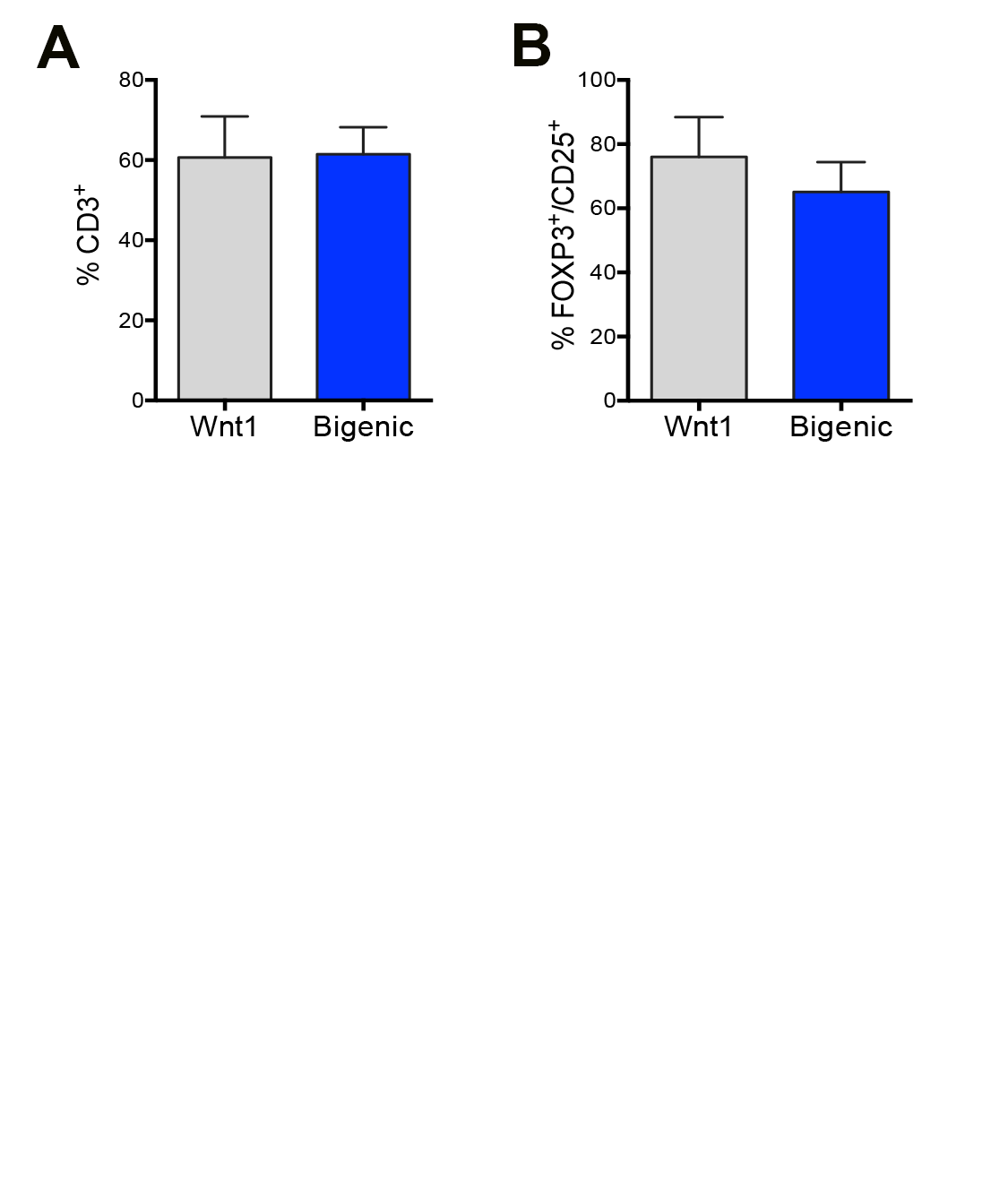

Supplement: Supplementary file 6 — Figure S3. Reduced IGF-1R in primary tumors does not alter the active T cell population. (A, B) Quantification of flow cytometry of CD3+ T cells (A) and activated regulatory T cells positive for FOXP3 and CD25 (B) in Wnt1 versus Wnt1/dnIGF-1R tumors (Wnt1/dnIGF-1R versus Wnt1 **P < 0.01; n = 20 each group). (TIF 70 kb) [file 13058_2018_1063_MOESM6_ESM.tif]

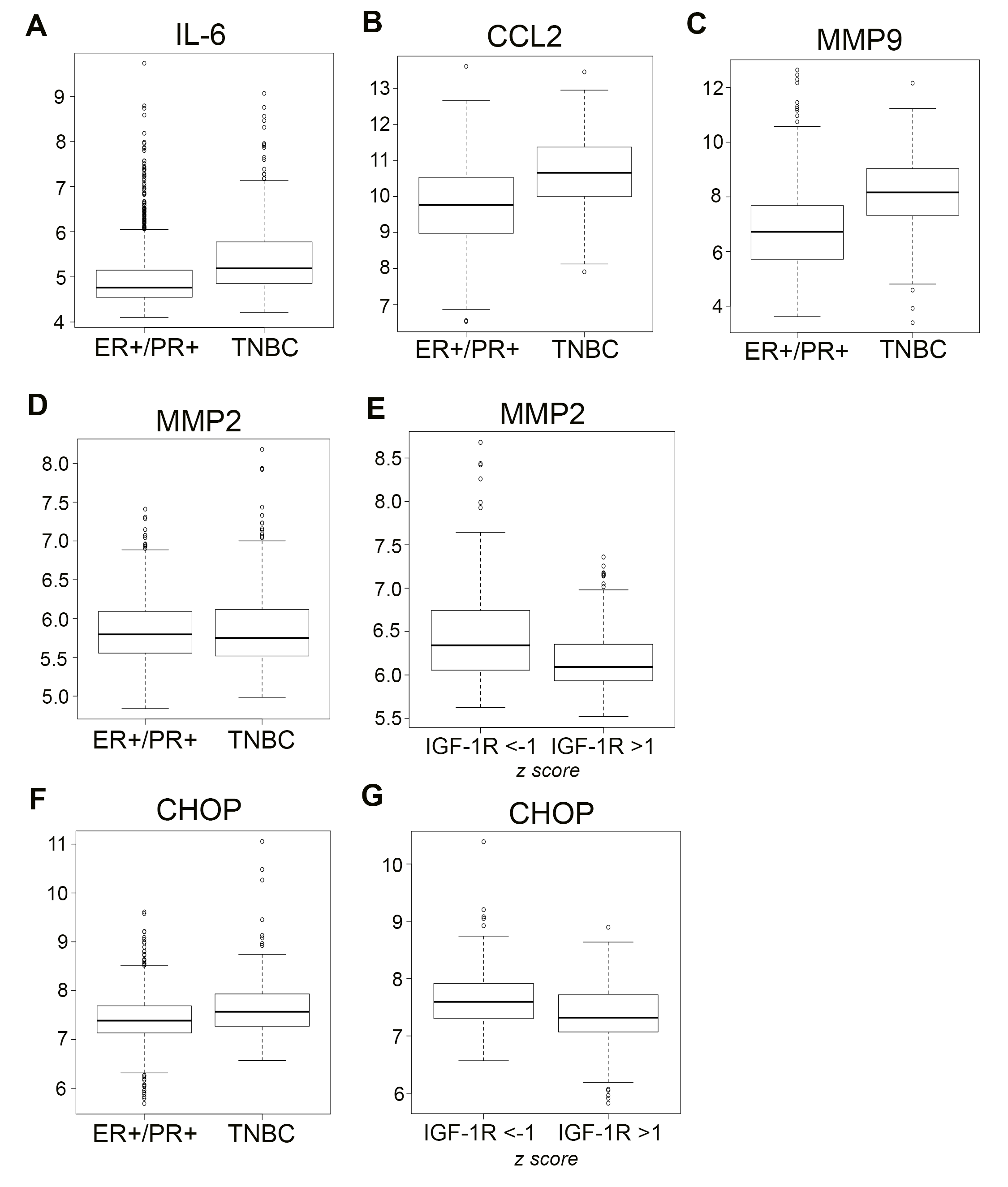

Supplement: Supplementary file 7 — Figure S4. Cytokine and MMP expression in human breast cancer patients. Analysis of gene expression from the METABRIC dataset. (A-C) Boxplot representation of IL-6 (A) (Student’s t test, P < 2.2 × 10− 16), CCL2 (B) (Student’s t test, P < 2.2 × 10− 16), and MMP9 (C) (Student’s t test, P < 2.2 × 10− 16) expression levels in ER+/PR+ breast cancer compared to triple-negative breast cancer (TNBC). (D-G). Boxplot representation of MMP2 and CHOP (D, F) expression levels in ER+/PR+ breast cancer compared to TNBC (MMP2: Student’s t test, P < 2.2 × 10− 16; CHOP: P < 2.2 × 10− 16). Boxplot representation of MMP2 and CHOP (E, G) expression in human breast tumors with low (IGF-1R z-score < − 1) versus high (IGF-1R z-score > 1) IGF-1R expression (Student’s t test, MMP2, P < 6.864 × 10− 10; CHOP, P < 3.172 × 10− 10). (TIF 253 kb) [file 13058_2018_1063_MOESM7_ESM.tif]

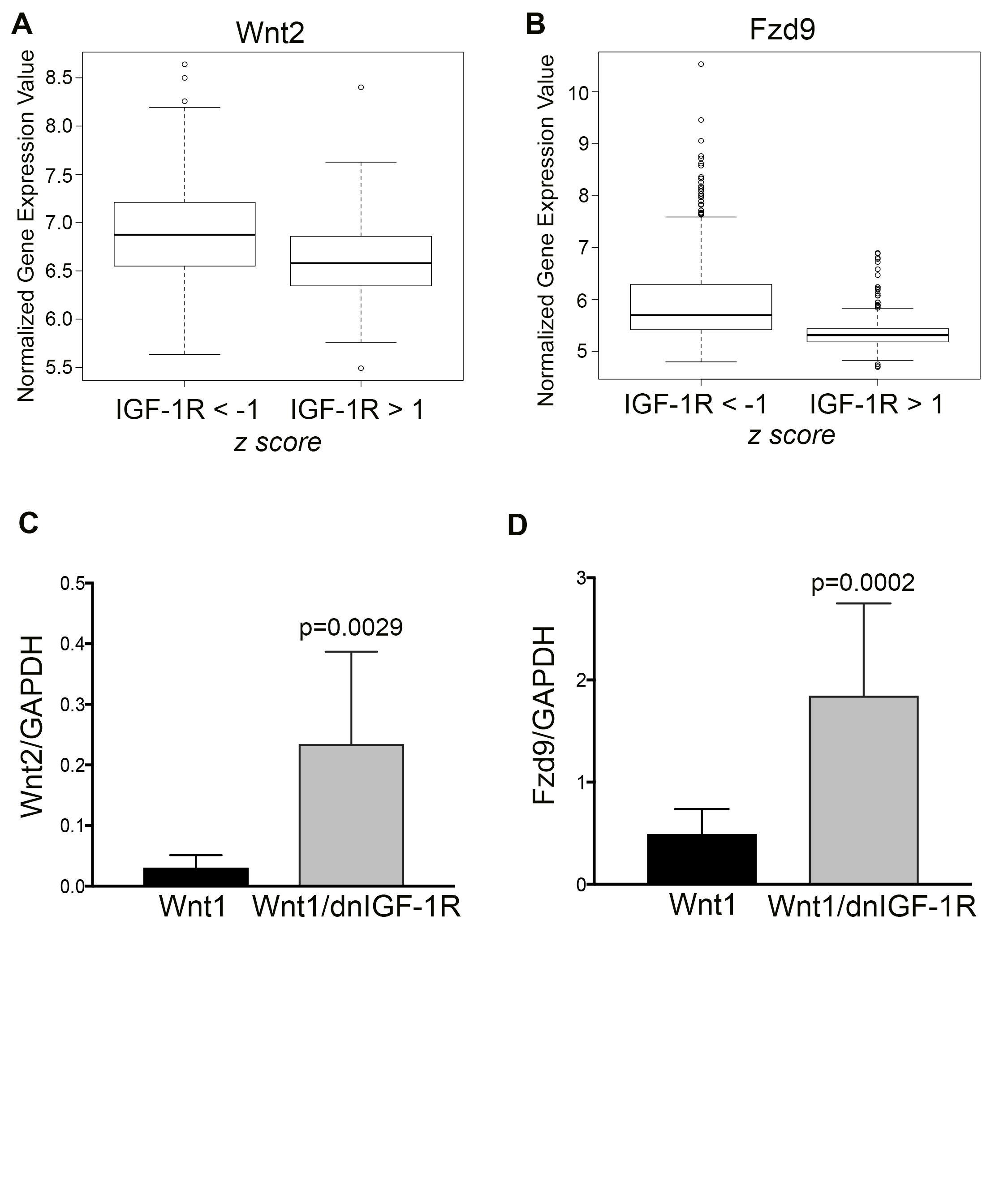

Supplement: Supplementary file 8 — Figure S5. Wnt2 and Frizzled9 expression are inversely correlated with IGF-1R expression in breast cancer. (A, B) Analysis of gene expression from the METABRIC dataset. Boxplot representation of Wnt2 (A) (Student’s t test, P < 2.2 × 10− 16) and Fzd9 (Frizzled9) (B) (Student’s t test, P < 2.2 × 10− 16) in human breast tumors with low (IGF-1R z-score < − 1) versus high (IGF-1R z score > 1) IGF-1R expression. (C, D). qRT-PCR analysis of Wnt2 (C) and Fzd9 (D) in MMTV-Wnt1 compared to MMTV-Wnt1/dnIGF-1R tumors (n = 4). (TIF 232 kb) [file 13058_2018_1063_MOESM8_ESM.tif]
