# Supplementary material for: Insulin-like growth factor receptor signaling in breast tumor epithelium protects cells from endoplasmic reticulum stress and regulates the tumor microenvironment
Source: Breast Cancer Res. 2018 Nov 20;20:138. doi: 10.1186/s13058-018-1063-2 (PMC6245538; doi:10.1186/s13058-018-1063-2)
Supplement: Supplementary file 4 — Table S3. Cytokine and chemokine profile is altered in MMTV-Wnt1 tumors with attenuated IGF-1R. Gene expression fold change measured by the ∆∆Ct method comparing MMTV-Wnt1 and MMTV-Wnt1/dnIGF-1R tumors. Student’s t test was performed to determine the corresponding p values. (DOCX 25 kb) [file 13058_2018_1063_MOESM4_ESM.docx]

| Gene Target | Fold change | p-value |  | Gene Target | Fold change | p-value |
| --- | --- | --- | --- | --- | --- | --- |
| AITR | 0.96 | 0.7641 |  | IL13R | 1.25 | 0.5485 |
| Ang1 | 1.01 | 0.6691 |  | IL15R | 0.55 | 0.0488 |
| Ang2 | 0.96 | 0.6976 |  | IL17RA | 0.73 | 0.2260 |
| April | 0.50 | 0.0394 |  | IL17RD | 1.49 | 0.2883 |
| CCL1 | 0.66 | 0.1491 |  | IL20R | 1.29 | 0.5494 |
| CCL2 | 2.54 | 0.0259 |  | IL21R | 1.39 | 0.1382 |
| CCL3 | 0.83 | 0.5194 |  | IL33R | 10.43 | 0.0125 |
| CCL4 | 1.41 | 0.0461 |  | IFNG | 0.83 | 0.3002 |
| CCL19 | 2.60 | 0.0649 |  | KitLig | 1.19 | 0.7264 |
| CCL20 | 0.64 | 0.2708 |  | LIFR | 0.58 | 0.0451 |
| CCR1 | 1.08 | 0.8522 |  | LTB | 0.93 | 0.6865 |
| cKit | 0.85 | 0.4075 |  | LTBR | 0.52 | 0.0039 |
| cMAF | 3.83 | 0.0777 |  | PRL | 3.01 | 0.0444 |
| CSF1 | 0.93 | 0.6643 |  | TGFB1 | 0.91 | 0.3428 |
| CSF2 | 0.58 | 0.2021 |  | TNFa | 0.75 | 0.2569 |
| CXCL1 | 0.21 | 0.0075 |  | TNFR1 | 0.80 | 0.2633 |
| CXCL2 | 0.20 | 0.0785 |  | TNFR16 | 1.26 | 0.5511 |
| CXCL3 | 0.74 | 0.3750 |  | VEGFA | 0.90 | 0.5657 |
| CXCL10 | 1.10 | 0.8732 |  | VEGFR1 | 1.36 | 0.3715 |
| GH | 5.55 | 0.0028 |  | VEGFR2 | 1.57 | 0.2186 |
| IL1B | 0.33 | 0.1402 |  | VEGFR3 | 1.07 | 0.9999 |
| IL3 | 0.58 | 0.2307 |  |  |  |  |
| IL4 | 1.50 | 0.1223 |  |  |  |  |
| IL5 | 0.60 | 0.2484 |  |  |  |  |
| IL6 | 3.37 | 0.0006 |  |  |  |  |
| IL7 | 0.69 | 0.0971 |  |  |  |  |
| IL9 | 4.75 | 0.0194 |  |  |  |  |
| IL10 | 3.54 | 0.0003 |  |  |  |  |
| IL11 | 1.79 | 0.0944 |  |  |  |  |
| IL12 | 3.91 | 0.0273 |  |  |  |  |
| IL13 | 1.15 | 0.6871 |  |  |  |  |
| IL19 | 3.18 | 0.0871 |  |  |  |  |
| IL21 | 3.77 | 0.0015 |  |  |  |  |
| IL23 | 0.41 | 0.0230 |  |  |  |  |
| IL31 | 2.59 | 0.0588 |  |  |  |  |
| IL33 | 5.78 | 0.0001 |  |  |  |  |
| IL1R | 2.05 | 0.0343 |  |  |  |  |
| IL2R | 0.60 | 0.1170 |  |  |  |  |
| IL4R | 0.81 | 0.3122 |  |  |  |  |
| IL6R | 0.42 | 0.0290 |  |  |  |  |
| IL7R | 0.91 | 0.6673 |  |  |  |  |
| IL9R | 4.32 | 0.0095 |  |  |  |  |
| IL11R | 1.18 | 0.7045 |  |  |  |  |
| IL12R | 0.35 | 0.1021 |  |  |  |  |

**Table S3.** Cytokine and chemokine profile is altered in *MMTV-Wnt1* tumors with attenuated IGF-1R.
